# Conceptual Model-Based Systems Biology: Mapping Knowledge and Discovering Gaps in the mRNA Transcription Cycle

**DOI:** 10.1371/journal.pone.0051430

**Published:** 2012-12-20

**Authors:** Judith Somekh, Mordechai Choder, Dov Dori

**Affiliations:** 1 Faculty of Industrial Engineering and Management, Technion, Israel Institute of Technology, Haifa, Israel; 2 Engineering Systems Division, Massachusetts Institute of Technology, Cambridge, Massachusetts, United States of America; 3 Faculty of Medicine, Technion, Israel Institute of Technology, Haifa, Israel; The John Curtin School of Medical Research, Australia

## Abstract

We propose a Conceptual Model-based Systems Biology framework for qualitative modeling, executing, and eliciting knowledge gaps in molecular biology systems. The framework is an adaptation of Object-Process Methodology (OPM), a graphical and textual executable modeling language. OPM enables concurrent representation of the system's structure—the objects that comprise the system, and behavior—how processes transform objects over time. Applying a top-down approach of recursively zooming into processes, we model a case in point—the mRNA transcription cycle. Starting with this high level cell function, we model increasingly detailed processes along with participating objects. Our modeling approach is capable of modeling molecular processes such as complex formation, localization and trafficking, molecular binding, enzymatic stimulation, and environmental intervention. At the lowest level, similar to the Gene Ontology, all biological processes boil down to three basic molecular functions: catalysis, binding/dissociation, and transporting. During modeling and execution of the mRNA transcription model, we discovered knowledge gaps, which we present and classify into various types. We also show how model execution enhances a coherent model construction. Identification and pinpointing knowledge gaps is an important feature of the framework, as it suggests where research should focus and whether conjectures about uncertain mechanisms fit into the already verified model.

## Introduction

A myriad of detailed pieces of knowledge regarding the structure and function of the living cell have been accumulating at an ever increasing rate while emphasis in biological research has shifted from probing into a single molecular function to studying complete cellular pathways, cycles and the entire cell as a system. For example, recent knowledge links the gene expression system stages (mRNA transcription, translation, and decay) by a single multi-functional heterodimer, named Rpb4/7, which we previously proposed to coordinate all stages into a system [1]. Thus, in order to better understand the expression of protein-encoding genes, we need to consider the entire multi-stage process, as each stage can be regarded as a subdivision of a continuous cyclical gene expression process. This realization calls for adopting a holistic, integrative, Conceptual Model-based Systems Biology that would enable making mechanistic system-level sense of the countless pieces of information that have been gathered thanks to decades of meticulous laboratory research by many thousands of scientists. A highly expressive conceptual modeling approach is needed not only for supporting researchers in integrating the knowledge, but also in gradually fleshing it out to see the “big picture”—the holistic view of a unified system.

In this paper we propose a framework for concurrently modeling structural and behavioral aspects of molecular biology systems and address the challenges of a coherent mechanistic model construction, its execution, and related knowledge gaps discovery and elicitation.

Molecular biology models that represent complex systems or subsystems may become very large, as they include many objects—proteins and other molecules and biocomplexes, and hierarchically organized processes. Constructing a biological mechanistic model can be compared to an attempt at assembling a huge jigsaw puzzle from an enormous number of parts—the known facts, many of which are not in a specific context, making the full picture incomplete or inconsistent. Conceptual approaches supporting a consistent unification of the qualitative facts regarding the mechanisms underlying the biological system are needed. These approaches must be expressive enough to address the various aspects of molecular biology systems. Moreover, the mounting facts constitute an impediment that renders purely manual model construction very tedious, time-consuming, and virtually impractical. Thus, automated construction of a large-scale mechanistic model from published research papers text using natural language processing technologies seems to be a viable option. However, the starting point for the automated model construction must be a kernel of the system under investigation that was manually-constructed, executed, and verified by a team of human experts that comprises system biologists and modeling experts.

As an underlying approach for conceptual modeling, the proposed framework adopts Object-Process Methodology (OPM), a holistic and graphical modeling language and methodology. Using a minimal set of generic, universal concepts—stateful objects and processes that transform them—OPM enables the representation of a rich set of abstractions of biological structures and behaviors. These abstractions provide for a consistent representation of knowledge about the functional, static, and dynamic aspects of biological systems at a spectrum of interconnected levels of abstraction, from molecules through organelles to the entire cell and its environment. A unique important feature of OPM models is that they are automatically translated on the fly into Object-Process Language (OPL), a set of natural English sentences in predefined templates that reflect all the details in the graphical model.

We take advantage of the relative simplicity of OPM and the fact that OPM models can be executed for analyzing complex biological system, understanding them, identifying model inconsistencies and knowledge gaps and classifying them, as the mRNA transcription case study presented in this paper clearly demonstrates. We also present the adaptations and modeling templates of OPM for molecular biology systems and demonstrate their utilization on the transcription case study.

### 1. Related Work

Specification and modeling of the dynamics biological systems, such as metabolic pathways, cell transduction, and regulatory networks, is currently carried out using a variety of methods [2], [3]. These modeling approaches can be roughly divided into (1) quantitative, mathematical equation-based approaches, such as Ordinary Differential Equations (ODEs) that describe the continuous variations in the concentration of substances and used in various environments [4], [5] or discrete stochastic approaches such as Gillespie's stochastic simulation algorithm [6] and (2) executable-qualitative approaches. Executable-qualitative approaches are used when data about quantities (e.g., concentrations) is missing, where equation-based approaches cannot be used. As our focus in this work is qualitative dynamic modeling aspects of biological systems, we briefly overview pertinent approaches, present their advantage and disadvantage, and compare their advantages and disadvantages to those of our proposed OPM approach.

Executable approaches for modeling biological systems use formal computational descriptions or algorithms to describe and understand natural phenomena [2]. **Boolean Networks** [7], [8] are graphs that include nodes and arcs between them, which describe gene regulatory and metabolic networks, focusing on cause and effect relationships among molecules or genes. In spite of their success in understanding concepts underlying biological systems, such as analyzing system robustness and steady states [7], [8], these network models are limited to Boolean effects of genes. They specify neither hierarchies nor details of the relevant molecules and processes involved in the system. OPM, on the other hand, has inherent, built in mechanisms for modeling both process and object hierarchies and present them at various levels of detail.

The most comprehensive works have used **Petri Nets** for modeling concurrent biochemical pathways [9], [10], [11], [12]. This established mathematical and graphical technique abstracts systems dynamic by tokens moving in a graph composed of arcs and nodes. The execution semantics of OPM resembles the concurrent execution semantics of the Petri-nets approach. Focusing on processes, Petri Nets do not easily lend themselves to modeling structural aspects such as molecules and their states, complexes, and molecular hierarchies. The **System Biology Graphical Notation** (SBGN) project [13], [14] aimed at standardizing a graphical representation of the biological model includes three types of graphical diagrams: process diagrams, entity relationship diagram, and activity flow diagram, inspired by Petri Nets. Each diagram type has distinct semantics for representing a biological system and provides a partial view of the overall system, making it quite difficult to mentally combine the diagrams into one holistic representation. Conversely, in OPM, the structure, behavior, and function of the modeled system are specified concurrently in a single holistic diagram type at various detail levels, preventing clutter and inconsistencies that may arise from using separated views for the various system aspects.

**Statecharts** is a formal graphical approach based on state transitions that defines the behavior of reactive objects over time. Statecharts-based models at the cell level and upwards were developed to describe the various stages in the life span of various cell types [15], [16]. Vulval cell fate determination in *C. Elegans* was modeled using Statecharts, expressing the mechanistic model, along with a scenario-based visual language called **Live Sequence Charts** (LSC), for modeling the experimental knowledge [17], [18], [19]. In Statecharts, a molecule may be represented by a state machine showing its possible states and event-driven transitions among them. OPM resembles the Statechart approach by being a qualitative executable approach, and lacks the ability to incorporate quantitative mathematical equations, that includes continuous or stochastic data. However, being state-oriented rather than process-oriented, as OPM is, reasoning about complex processes, in which many types of molecules take part at various refinement levels, is not straightforward. Molecular transient structures, such as complex formation, which are easily modeled with OPM, are not straightforward to model in Statecharts either. While Statechart model execution is driven by (optionally conditioned) state changes in response to asynchronous events, the OPM mix serial and concurrent scenario execution. In OPM, each process can have conditions that limit its execution. To model quantitative behavior, in both methods, multiple instances for each biological object can be added and the system behavior can be then executed and analyzed [15].

**Process Algebras** are formal languages for specifying systems with discrete events. For example, Regev et al. [20] proposed to represent biochemical signaling pathways through the use of process algebra language, the π-calculus, originally developed to model networks of communicating processes. Using this approach, communication was mapped to molecular binding processes, and channels were mapped to the binding domains of these biomolecules. These languages are concurrent and compositional, but being text-based only, they are less intuitive than graphical or bi-modal representations, such as the bi-modal graphics-and text model representation of OPM. In **Agent-based** approaches, computational entities called agents execute their tasks autonomously and concurrently. A biological system is modeled as a set of agents in a dynamic and often unpredictable environment that interact through the creation or modification of signals on a shared data structure, known as a “blackboard” [21]. Applying an Object-Oriented (OO) Unified Modeling Language (UML)-based and agent-based approach, Webb and White [22] modeled and simulated metabolic and genetic pathways, using Statecharts and message exchange. Due to limitations of the OO paradigm that stem from its origins in the software domain, this model includes such non-biological artifacts as capsules, ports, and connectors that exchange messages, making it less than intuitive.

It is worth mentioning the **Rule-based** approach [23], [24] which is another method for dealing with incomplete quantitative data. The approach has the ability to represent rich variety of biological knowledge regarding structure, behavior and experimental external conditions. The formalisms consists a set of facts and a set of rules (with condition and action parts), stored in a knowledge base. Rule-based simulation, iteratively matches the facts in the knowledge base against the condition parts of the rules, and executes the matched action parts. Facts can express a rich variety of knowledge about the objects of the biological system, which can represent molecule, biological process or environmental conditions. The objects are usually hierarchically structured and are described by their attributes. BIOCHAM [24] implements a rule-based approach for model specification which is complemented with a temporal logic language for the verification of the properties of the biological models. Although the advantage of the rule-based approach to represent rich variety of qualitative and quantitative knowledge, it is a text-based approach which makes it less than intuitive for humans. OPM can also specify a rich variety of biological knowledge, such as biological objects, their attributes, their states, biological processes, process and object hierarchies and environmental conditions (as we demonstrate in the sequel), but unlike rule based approaches, which are textual, OPM is graphical.

With respect to the Semantic Web and its capabilities, the Visual Semantic Web (ViSWeb) is a paradigm for enhancing the current Semantic Web technology [25] that is based on OPM. ViSWeb enables modeling of systems in a single graphic and textual model, providing for representation of knowledge over the Web in a unified way that caters to human perceptions while also being machine processable. The advantages of the ViSWeb approach include equivalent graphic-text knowledge representation, visual navigability, semantic sentence interpretation, specification of system dynamics, and complexity management. Arguing against the claim that humans and machines need to look at different knowledge representation formats, the principles and basics of various graphic and textual knowledge representations are presented and examined as candidates for ViSWeb foundation. Since OPM is shown to be most adequate for the task, ViSWeb is developed as an OPM-based layer on top of XML/RDF/OWL to express knowledge visually and in natural language. Both the graphic and the textual representations are strictly equivalent. Being intuitive yet formal, they are not only understandable to humans but are also amenable to computer processing. The ability to use such bimodal knowledge representation is potentially a major step forward in the evolution of the Semantic Web.

Although the methods briefly surveyed above are appropriate for computational analysis of various aspects of the system under study, most of them abstract only part of the information regarding the biological system such as hierarchical structures, variable states, system events and behavioral details of the molecular biology system. As we show next, OPM has an advantage of being able to holistically integrate most of the biological information types and concurrently model and execute models of complex molecular biology systems. We note upfront that in its current form, OPM is a qualitative executable approach, and it lacks the ability to incorporate continuous or stochastic data into its models. We discuss this aspect in more detail in the sequel.

### 2. Object-Process Methodology

Object-Process Methodology [26] is a holistic graphical approach to the representation and development of complex systems while maintaining a formal framework. OPM was originally aimed for use by systems engineers for knowledge management and representation of multidisciplinary man-made socio-technical industrial and information systems [26]. OPM is founded upon two elementary building blocks. These are stateful objects - things that exist, such as molecules, which represent the system's structure, and processes - things that happen to objects and transform them. Processes transform the system's objects by creating them, consuming them, or changing their states. Two semantically equivalent modalities, one graphic and the other textual, are used to describe each OPM model. The graphical model is automatically translated into a textual model. The textual representation which is built as a subset of English can ease the comprehension of the models by non-expert viewers.

**Figure 1:**
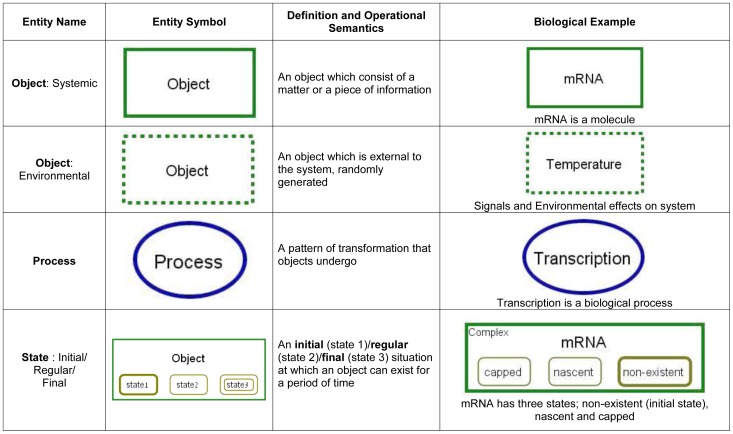
OPM entities with their symbols, definitions and operation semantics.

**Figure 2:**
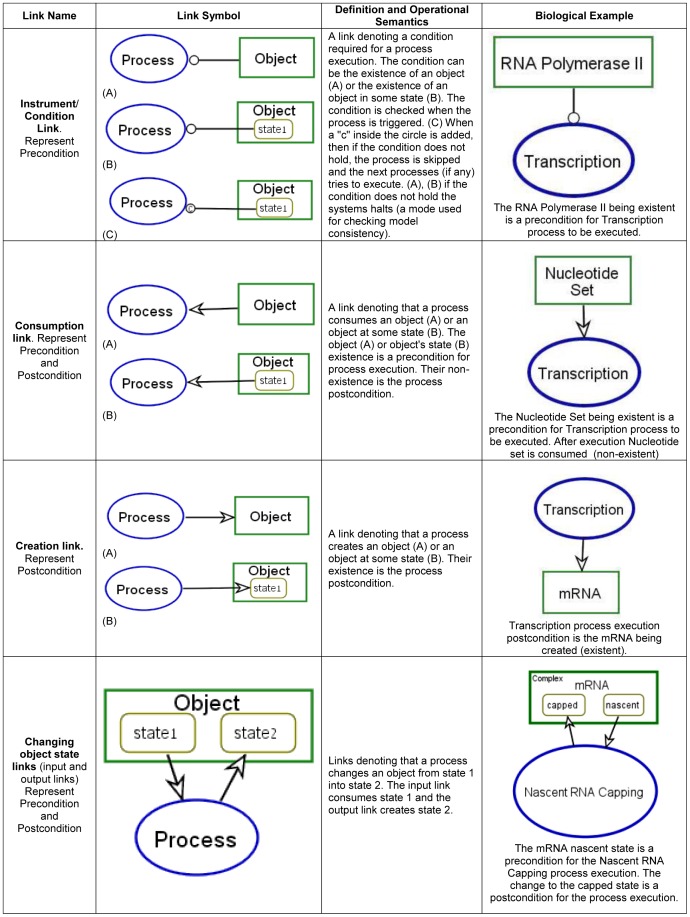
OPM procedural links: links connecting an object or state with a process. These links represent process pre/postcondition object set.

**Figure 3:**
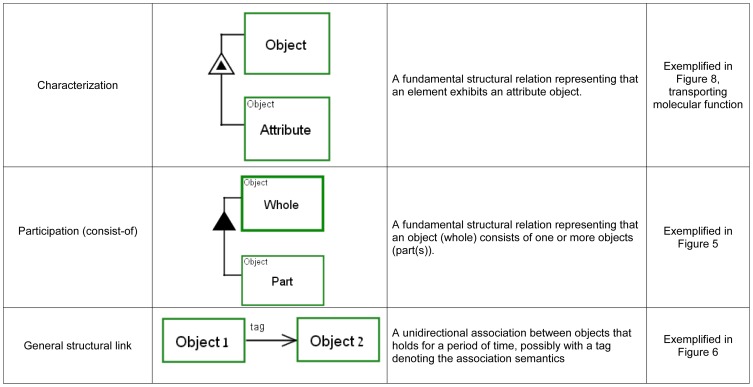
OPM structural links: links connecting an object with an object. Theses links represent structural hierarchies and characteristics.

By using a single holistic hierarchical model for representing structure and behavior, clutter and incompatibilities can be significantly reduced even in highly complex systems, thereby enhancing their comprehensibility. OPM has proven to be better in visual specification and comprehension quality when represented complex reactive systems and compared to the standard in the field of systems engineering [27]. OPM is supported by OPCAT [28], a software environment that is used in this work to model the transcription case study presented later in this paper. OPM operational semantics were recently defined by a translation into a state transition system [29], [30] and a related OPCAT simulation environment was developed [28]. OPM main elements with their semantics and biological examples are presented in Figure 1, Figure 2 and Figure 3 (for full semantics see [26]).

#### 2.1. OPM operational semantics

The OPCAT simulation environment supports concurrent, synchronous and discrete time execution. The execution we used in this work is qualitative in nature with one instance defined for each object (e.g., molecule) and process. This enables detecting model errors by executing and analyzing the qualitative mechanisms underlying the biological system under study. While multiple instances can be defined in OPCAT simulation and quantitative aspects can be inspected, these are out of the scope of this work.

Processes are executed in a synchronous manner, one after another according to a defined timeline. The default timeline, within the context (in-zoomed frame) of each process, is from top to bottom. Alternative scenarios or loops, which override the default timeline, can be modeled using an invocation link (see Table S1). Concurrency is supported, and processes whose ellipse topmost points are located at the same height in the diagram are being executed concurrently.

Each process has a (possibly complex) precondition and a postcondition. A process is triggered (attempted to be activated according to its place in the timeline), and its precondition is then checked. Only if the precondition is satisfied, the process is executed. Upon normal process termination, the postcondition must hold. The precondition of a process is expressed by its preprocess object set—the set of objects, which must exist, some possibly in specific states, for the process to start. The postcondition is defined similarly by the postprocess object set. Figure 2 exemplifies the links for modeling objects and states as process preconditions and postconditions. Logical expressions (AND, OR, XOR) between objects in the pre- and post-process object set can be defined (see Table S1). By default, the logical relation between the objects in the pre-process or post-process object set is a logical AND, meaning that all the objects in the preprocess object set must exist in their defined states for the process precondition to be true. It is possible to change this default definition by using the XOR and OR relation between various objects. Process execution can also depend on random signals. To model this, we connect the process to an environmental object (see Figure 1) without or with a specific state. This environmental object is added to the preprocess object set, i.e., process pre-conditions. OPM semantics also include event links, for modeling reactive systems, and time exception links which are not used in our biological models. The complete OPM semantic is specified in [26].

During execution initiation, system objects are initiated to be at state “existent”. Objects created later during execution are initiated to be “non-existent”. All objects with explicit states are initiated to their initial state, or, if not defined, to a random state. Environmental objects are randomly chosen to be existent or non-existent. If an environmental object is stateful (has states), one of its states is randomly chosen. OPM formal operational semantics can be found in [30].

#### 2.2. Handling system complexity via in-zooming and unfolding

The complexity of systems is managed in OPM models by abstraction-refinement mechanisms, notably out- and in-zooming and folding/unfolding, which is used to hierarchically expose or hide details of processes and objects (e.g., molecules), respectively. This way, a top-level view of the system is expanded into a set of increasingly detailed diagrams that provide the details of the processes (via in-zooming) and objects (via unfolding) shown in the top-level view. These two mechanisms, process in-zooming and object unfolding, are done simultaneously during model construction. While zooming into processes, the structural or characteristic details of objects are exposed via unfolding. Three of OPM's structural object-object relations are used in this work: the aggregation-participation (“part-of”) relation, the exhibition-characterization (“attribute”) relation and the general unidirectional relation (shown in Figure 3).

#### 2.3. Query processing capabilities

OPM has the following query processing capabilities, embedded in OPCAT. (1) Find: one can do a simple “find” query and get the list of all places in which a particular string of characters appears in the OPM model. This can be filtered by objects, processes, or states, and can be searched as a string or as a regular expression. In response OPCAT provides a table with all the found locations. Clicking on each line takes the user directly to the relevant OPD and highlights the object, process, or states in red. (2) “Show All Appearances”: right clicking on a thing (object or process) provides a table with all the found locations. Clicking on each line takes the user directly to the relevant OPD and highlights the object or process in red. (3) XQuery: Since OPL can be extracted as XML, it can be directly queried by using XQuery [31], a query and functional programming language that is designed to query collections of XML data.

**Figure 4:**
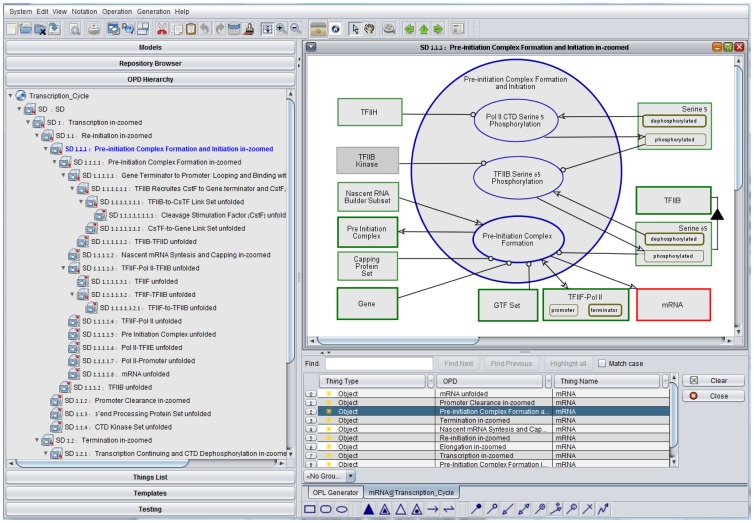
An example of OPM query capability: mRNA search.

Figure 4 shows an example of OPM query capability, where the object mRNA search was done using “Show All Appearances”, providing in response the table at the bottom right of Figure 4 with nine appearances of mRNA in various OPDs in the model. Clicking the third line of the table brought us to the OPD titled “**SD1.1.1. Pre-initiation Complex Formation and Initiation in-zoomed**”, in which mRNA is highlighted in red. The OPD tree on the left pane shows part of the OPD hierarchy resulting from recursively zooming into yet lower-level processes. In this pane, SD1.1.1 is highlighted in blue to show where we are in the OPD tree.

Having investigated the expressiveness of OPM in its current form to model molecular systems, we have found that OPM lacks dedicated patterns for modeling the full range of biological structures and behaviors, such as link hierarchies and transient relations, forming complexes among biological entities and various molecular functions. In this paper we explore the characteristics of molecular biology systems from a conceptual qualitative modeling viewpoint and classify molecular functions. We expand OPM to accommodate these modeling constructs, and evaluate the effectiveness of the developed modeling framework. The adaptations and templates are evaluated by applying them to model the mRNA transcription cycle, a subsystem of the gene expression system. Through construction and qualitative execution of the resulting model using OPCAT tool, we show how modeling errors and knowledge gaps are identified and we classify them into several types for the purpose of assisting in the generation of wet laboratory experiments aimed to close these gaps.

## Results

### 1. OPM Adaptations for Molecular Biology Systems

A valuable qualitative model of a biological system should represent its static, dynamic, and functional views [12]. As we demonstrate below, a single OPM diagram type supports these three major system aspects, relieving us from the need to use three or more different diagram types for these three aspects, thereby avoiding the need to try to understand the overall view of the system by collecting and mentally combining details from disparate diagram types. OPM has a compact set of conceptual building blocks for representing holistically these three aspects.

#### 1.1 Modeling biological complex structures

Biological objects vary in size, starting from single molecules of growing size through molecular complexes, all the way to the more complex structures, including organelles, cell compartments, and the cell as a whole system. Our focus is modeling of molecules, complexes, and interactions among them; higher-level biological objects, which include multi-cell organisms, societies of organisms, and entire ecological habitats, are beyond the scope of this work. In this section we focus on modeling molecular structures and associations between molecules. In the following sections we present modeling templates of molecular functions and the formation of complexes.

Biological complexes are cellular components composed of molecules (e.g., proteins), which are often further decomposed into several structural domains [32]. A complex can be composed of other complexes as well. An example of a complex is the transcription Pre-Initiation Complex, which is composed of other complexes, including the general transcription factors TFIIB and TFIIF. In humans, the complex TFIIF is composed of the protein Tfg1 and other proteins [33]. A *domain* is the protein's building block, and it has a distinct function [32]. In molecular evolution, domains are recombined in different arrangements to create proteins with different functions. A domain can interact with more than one molecule, and it can therefore include more than one binding site. A protein *binding site* is defined as the minimal region that is required to bind another molecule. A binding site is composed of some set of consecutive amino acids. One binding site can bind more than one pair of interacting partners, but not simultaneously. Two binding sites can be situated in different or partially overlapping 3D regions in the same domain.

**Figure 5:**
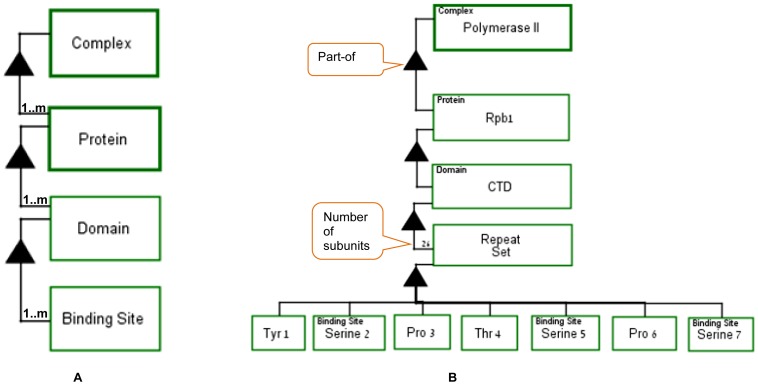
Complex generic model and example. (A) A generic model of the structure of a Complex. The object Complex consists of at least one (denoted by “1..m”) Protein, which consists at least one Domain, each of which, in turn, consists at least one Binding Site. (B) The Complex **Polymerase II** with one of its proteins, **Rpb1** and its 26 **Repeat Sets** with their structure. The balloons include explanation of the OPM semantics.

Based on these definitions of domain and binding site, Figure 5 presents a generic OPM template of the structure of a complex and an actual example. In Figure 5A, the object **Complex** consists of at least one (denoted “1..m” – one to many) **Proteins**. Each **Protein** consists of at least one **Domain**, each of which, in turn, consists of at least one **Binding Site**. Figure 5B exemplifies application of this template to the **Polymerase II** complex, which is the main transcription machinery. **Rpb1** is one of the 12 proteins composing **Polymerase II**. The C-terminal Domain (**CTD**) of **Rpb1** in *S.cerevisiae* is composed of 26 **Repeat Sets** of amino acids. **Serine 2**, **Serine 5**, and **Serine 7** are modeled as **Binding Sites**. The type of the object (e.g., Complex, Molecule (Protein), Domain, Binding Site) is denoted at the upper-left corner of each object and must correspond to the template, as can indeed be verified by comparing the template on the left to the example on the right.

**Figure 6:**
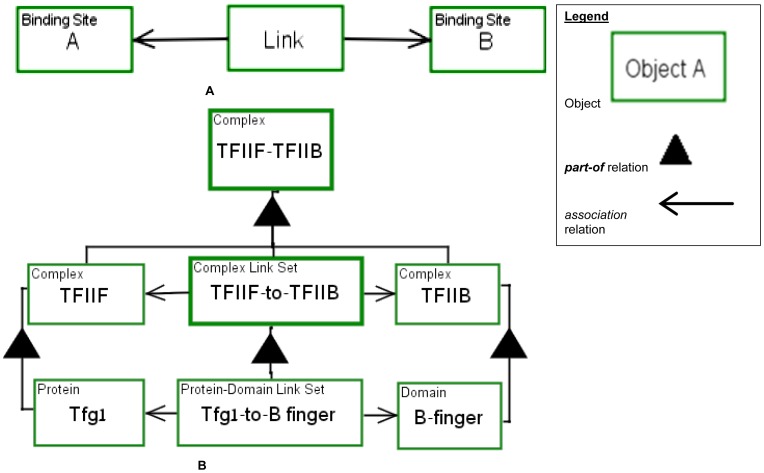
Generic link object and example. (A) A generic simple **Link** example. The object **Link** connects two binding sites A and B. The **Link** object can be created by a binding process and consumed by a dissociation process. (B) The **TFIIF-TFIIB** Complex is composed of a **TFIIF** Complex, a **TFIIB** Complex and a **TFIIF-to-TFIIB** Complex Link Set.

As Figure 5 shows, biological structures and the associations between them are complex and often hierarchical. They should be expressed with the ability to refine structures and links at several levels of abstraction, ultimately revealing the most basic elements – the binding sites. To this end, our framework provides a hierarchy of structures and a corresponding hierarchy of links. In order to model hierarchical molecular associations clearly and explicitly, we define **Link** as a specialized OPM object that represents the association between two molecules. As Figure 6A shows, **Link** is connected to two Binding Sites via the OPM unidirectional structural link—an open head arrow. **Link** associations are by default non-covalent. Covalent associations are modeled with one of the catalyzing templates presented in the following section.

The **Link** object provides for creating a link hierarchy. **Link** is the lowest object in the link hierarchy. Above it is the **Domain Link Set**. As defined in Figure 5, a **Domain** is composed of a set of **Binding Sites**. Two domains are linked via a **Domain Link Set** object—a set of one or more **Links**, each associating two **Binding Site** objects. Analogously, one level up the link hierarchy, two proteins, each consisting of one or more **Domains**, are associated via a **Protein Link Set** object—a set of one or more **Domain Link Sets**. At the top level, two complexes, **Complex** objects, are connected by a **Complex Link Set** object.

As an example for modeling biological structures consider the following sentence, cited from [33]:

*“Tfg1, the largest subunit of TFIIF, [is] also cross-linked with the B-finger and linker domains, demonstrating a close association between Tfg1 and these domains of TFIIB”*.

Figure 6B presents a model of the hierarchical association between the TFIIB and TFIIF complexes via the B finger domain and the Tfg1 subunit. The **TFIIB-TFIIF** Complex is composed of the **TFIIB** Complex and the **TFIIF** Complex, connected by **TFIIF-to-TFIIB** Complex Link Set. The **TFIIF-to-TFIIB** Complex Link Set is further decomposed into its set of links, the **Tfg1-to-B-finger** Protein-Domain Link Set. The **Tfg1-to-B-finger** Protein-Domain Link Set represents the binding between the **B-finger** domain of **TFIIB** and the **Tfg1** subunit of **TFIIF**. This **Tfg1-to-B-finger** Protein-Domain Link Set connects the respective parts of the **Tfg1** Protein and the **B-finger** Domain. If the finer structure is known, the domains can be further decomposed into their binding sites, and then the actual links comprising the **Tfg1-to-B-finger** Protein-Domain Link Set can be specified in the model. Since the type of each object is recorded in the top-left corner of each object box, we can tell, for example, that **TFIIF–TFIIB** is a Complex, so its name is **TFIIF-TFIIB** Complex, and **Tfg1** is a Protein, so its name is **Tfg1** Protein. Alternatively, declaring Protein and Complex to be reserved words in our framework, we can call the two objects “The Complex **TFIIF–TFIIB**” and “The Protein **Tfg1**”.

#### 1.2 Modeling biological molecular functions

According to the Gene Ontology, GO [34], [35], *a biological process is accomplished via one or more ordered assemblies of molecular functions*. Adopting this definition in our framework, *molecular functions* are a small set of basic, non-decomposable processes that transform biomolecules. Any higher level biological process is composed of these molecular function building blocks. This process hierarchy spans the spectrum ranging from the simple molecular functions all the way to the most complex biological processes, such as gene expression. This hierarchy is clearly represented by the tree of Object-Process Diagrams (OPDs) created top-down by recursively zooming into processes, starting from the high-level function (e.g., mRNA lifecycle), and ending with molecular functions as the tree leaves.

In GO, molecular functions are classified into four basic categories: non-covalent binding, enzymatic activity, receptor activity, and transporter activity. Inspired by this classification and building on our experience in modeling the mRNA transcription and decay, we classify molecular functions into the following three basic process classes.

**Catalyzing** – enzyme-based stimulation of a reaction, involving one or more molecule types. Catalyzing is further divided into Substrate Consumption Catalyzing and Substrate Change Catalyzing.

**Binding/Dissociation** – non-covalent interaction of a molecule X selectively with a molecule Y within the same cell compartment. Dissociation is the inverse of binding. We note that covalent interactions are included in the catalyzing molecular function.

**Transporting** – a directed movement of a molecule across cell compartments.

As noted, higher level biological processes are composed of these basic molecular functions. For example, shuttling of molecule M might involve *Binding* to molecule B, followed by *Transporting* the resulting B-M complex across a cell compartment boundary, followed by *Dissociation* of M from B.

**Figure 7:**
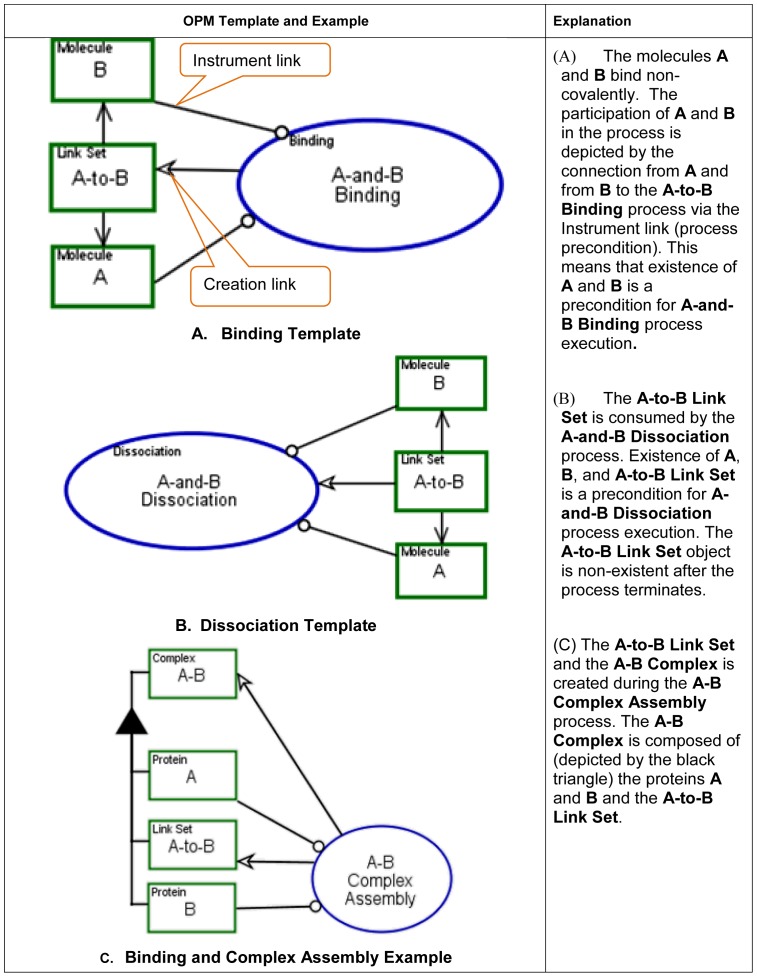
Binding/Dissociation molecular function, modeling templates and example.

**Figure 8:**
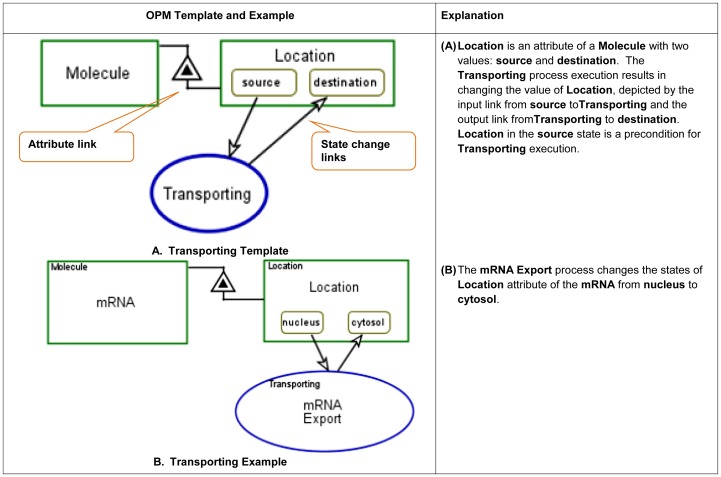
Transporting molecular function, modeling template and example.

**Figure 9:**
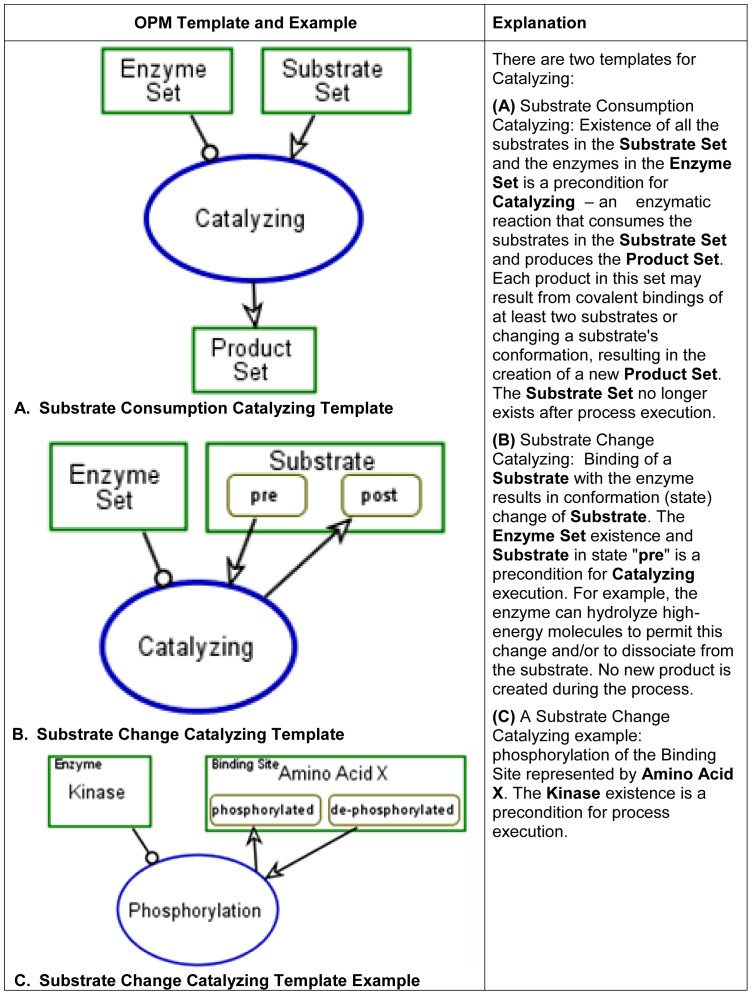
Catalyzing molecular function, modeling templates and example.

The OPM graphical modeling templates, examples and execution semantics for these three molecular functions are presented in Figure 7, Figure 8 and Figure 9.

In OPM, structure and behavior are combined in a single diagram type, representing explicitly how the system's behavior effects its structure. For example, applying the molecular binding template, example C in Figure 7, *Binding and Complex Assembly*, shows the process of binding two proteins **A** and **B** and the effect on the newly created **A–B** Complex. The biological objects are **A** Protein and **B** Protein, **A–B** Complex, and **A-to-B** Link Set. **A** Protein and **B** Protein participate in the **A–B Complex Assembly** process, along with the two newly created objects: **A–B** Complex and **A-to-B** Link Set. Their creation is represented by the creation links (arrows) emanating from the **A–B Complex Assembly** process to these two objects. The hierarchical structure is represented via the “part-of” (black triangle) structural link, connecting **A–B** Complex as a whole to its two parts, **A** protein and **B** protein. The details about the specific binding domain and binding sites can be further exposed using the in-zooming mechanism. A more detailed explanation on complex formation follows.

#### 1.3 Hierarchical associations and dissociations

The complex hierarchical details of molecular associations call for developing and using adequate modeling tools. Our mechanism for this purpose includes a link hierarchy that starts with general inter-complex links at the top, all the way down to inter-binding sites links. As we climb up the OPD set (set of OPM diagrams) tree to higher level diagrams (using the out-zooming mechanism), low-level details about the domains and binding sites become invisible. They are exposed only when we drill down and inspect increasingly detailed biological processes, and eventually molecular functions.

**Figure 10:**
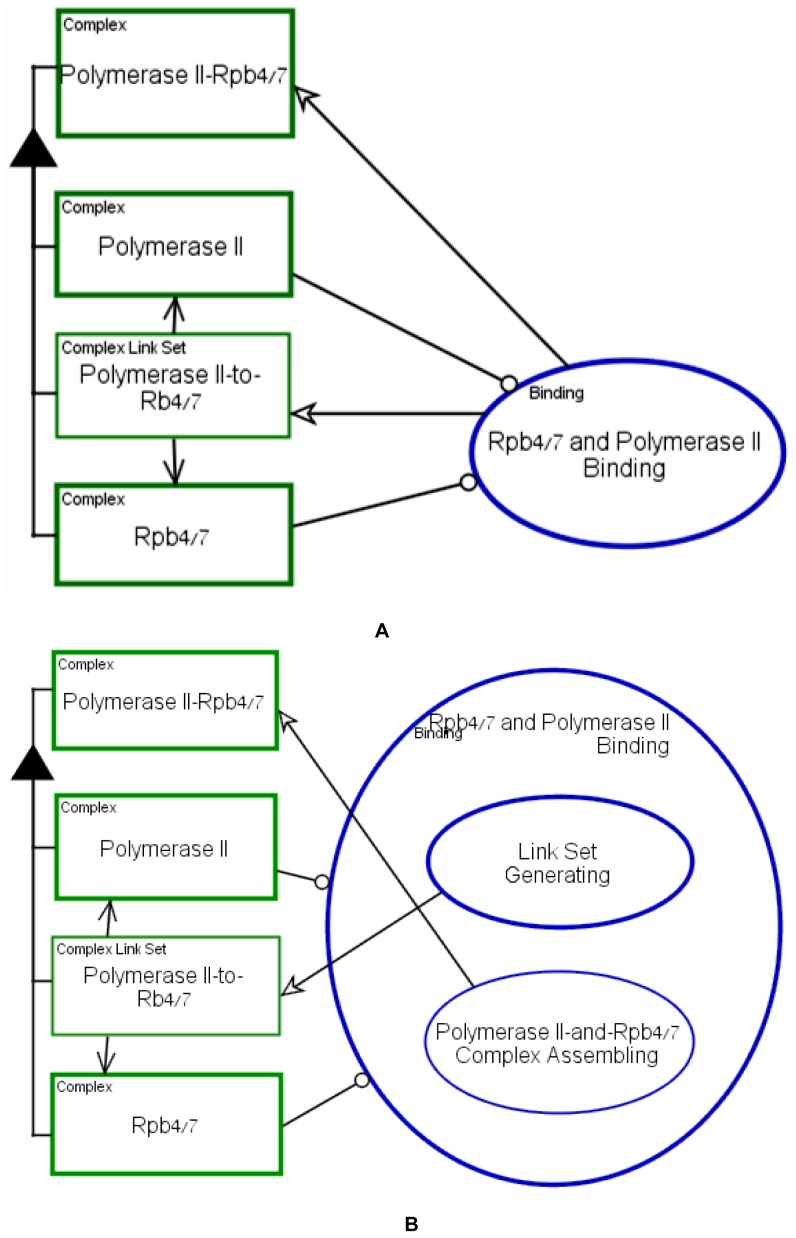
Complex formation: the process of molecular binding exemplified on Rpb4/7 to Polymerase II binding. (A) **Rpb4/7 and Polymerase II Binding** process (B) **Rpb4/7 and Polymerase II Binding** process zoomed into its sub-processes, **Link Set Generating** and **Polymerase II–and-Rpb4/7 Complex Assembling**.

A complex formation process is exemplifies in Figure 10 on Rpb4/7 binding to RNA Polymerase II. Rpb4/7 is known to be a subtoichiomertic component of RNA Polymerase II [1]. The two diagrams in Figure 10 show (A) the process of binding **Rpb4/7** and **Polymerase II**, the two complexes participating in the **Rpb4/7 and Polymerase II Binding**, and (B) details of this process. The created complex and links are shown concurrently. The details about the specific binding domain and binding sites can be further exposed through further in-zooming. The OPM diagram resulting from zooming into the **Rpb4/7 and Polymerase II Binding** process in Figure 10A is shown in Figure 10B. The exposed subprocesses are (1) **Link Set Generating**, which is further in-zoomed to expose the exact details of binding links and domains (not shown) and (2) **Polymerase II-and-Rpb4/7 Complex Assembling**, which creates the complex and connects it to its parts during model execution. It is up to the system modeler to decide what level of granularity is needed (or known) for the purpose of understanding some specific point about the system and the associated biomolecular mechanism.

In many cases, a protein-protein interaction is known to occur, but the exact domains or binding sites of this interaction is unknown. In such cases, we model the general protein-protein interaction, as was done in Figure 10A, without zooming further into the binding process and without exposing the binding domains or sites. This selective refinement enables modeling a system with unknown data, yet providing for executing it correctly.

#### 2. The Transcription Cycle Case Study

To evaluate the utility of OPM as a language for modeling molecular biology systems, we have modeled the mRNA transcription cycle. We present the OPM model of this system, its execution, the knowledge gaps detected as a result of this modeling process, and the classification of these knowledge gaps. In addition, by executing the model in a “halt execution” mode (i.e., halting whenever a process precondition is not satisfied), we show how the execution can detect model errors, which may result either from modeling errors or from actual knowledge gaps.

The expression of protein-encoding genes is a complex process that determines which genes are expressed as proteins at any given time, as well as the relative levels of these proteins. The mRNA Lifecycle involves several distinct stages: (1) RNA synthesis, or transcription and RNA processing (after which the RNA is considered mRNA), (2) mRNA transport (in eukaryotes) from the nucleus to the cytoplasm, (3) protein synthesis, or translation, and (4) mRNA degradation. RNA polymerase II (pol II), a large multi-subunit complex, is responsible for transcribing protein-encoding RNAs, namely mRNAs, which are the focus of our case study.

Transcription by pol II, the first stage in the expression of protein-encoding genes, produces RNA—the primary transcript. To initiate transcription, pol II requires a series of additional proteins, general transcription factors, and other proteins (e.g., activators). In addition, the Mediator, a large multiprotein complex, and the chromatin (which includes the DNA) with its main building blocks, the histones, are responsible for modulating transcription by communicating with many gene-specific regulators and transcription activation factors [36]. During the elongation phase of transcription, the nascent RNA undergoes three types of processing events: (I) a special nucleotide, m(7)GpppN, named “cap”, is added to the RNA's 5′ end (a process known as capping); (II) intron sequences are removed from internal sections of the RNA molecule (splicing) (III) A stretch of poly(A), called poly(A) tail, is added to the 3′ end of the RNA. This process involves RNA cleavage and further polyadenylation, and is executed prior the transcription termination phase, which occurs downstream of this site. Each of these processes is carried out by proteins or RNA molecules, many of which travel along with the RNA polymerase II (Pol II). In many cases, these modifying factors bind to C-terminal Domain (CTD) of Pol II. Transcription of a given gene is a multiple round event, during which RNA Polymerase II undergoes phase transitions between “initiation”, “elongation”, and “termination”, which can repeat many times. It has been proposed [37], [38], [39] that the same Pol II can be transformed from the termination to initiation phase without leaving the transcription unit. Thus, termination may be coupled to initiation. The first transcription round is a rare event compared to subsequent rounds that involve termination-coupled with re-initiation. Some of the initiation factors remain bound to the promoter throughout the transcription cycle, whereas others are recycled [37], [38]. Indeed, convincing recent evidence from both *in vitro* and *in vivo* studies have shown that general transcription factors (GTFs), such as TFIID, TFIIA, and TFIIB [37], [38], [40], [41], [42], as well as the Mediator complex [36], [38], [39], [43], stay behind at the promoter when Pol II engages in transcript elongation, allowing rapid entry of new polymerases for re-initiation of transcription at the gene. The Chromatin and Mediator roles, which are more significant in the first pioneering round of transcription [36], [38], [39], are beyond the scope of this paper.

Our transcription model, which yields an mRNA, includes RNA synthesis and processing. The model focuses on the transcription reinitiating process and its participants; the basal Pol II transcription machinery in eukaryotes, the general transcription factors TFIID, TFIIB, TFIIE, TFIIH [37], [38], [40], [41], Rpb4/7 [1] and Fcp1 [44]. Our main goal was to gain insight into the reinitiating process and the role of rpb4/7 in it. It should be emphasized that, although based on compelling results [37], [38], the looping model cannot yet be considered to be a well-established mechanism. We suspect that it is relevant to some transcription units, but not to all of them.

#### 2.1 Transcription OPM model

The OPM model and execution of this important cellular subsystem with illustrations of OPM extensions and templates is presented in this section. The transcription model is based on 32 facts and mechanisms derived from 19 research papers (presented in Table S2), regarding the mechanisms underlying the transcription process in eukaryotes. Our main focus was the transcription re-initiation process and its related factors: TFIIF transcription factor, RNA Polymerase II, its CTD(C-Terminal Domain) changes and its Rpb4/7 subunit, TFIIB transcription factor and Fcp1 phosphatase. Other participating transcription factors such as- TFIIA, TFIID, TFIIH, TFIIE where modeled as well, whenever related to the re-initiation process.

The established model includes 50 objects and 37 processes. The 37 processes includes, 13 higher level processes and 24 lowest level processes. Lowest level processes are not further in-zoomed. The model's processes tree includes 7 levels. The transcription model and the OPCAT tool can be downloaded and executed from [45].

**Figure 11:**
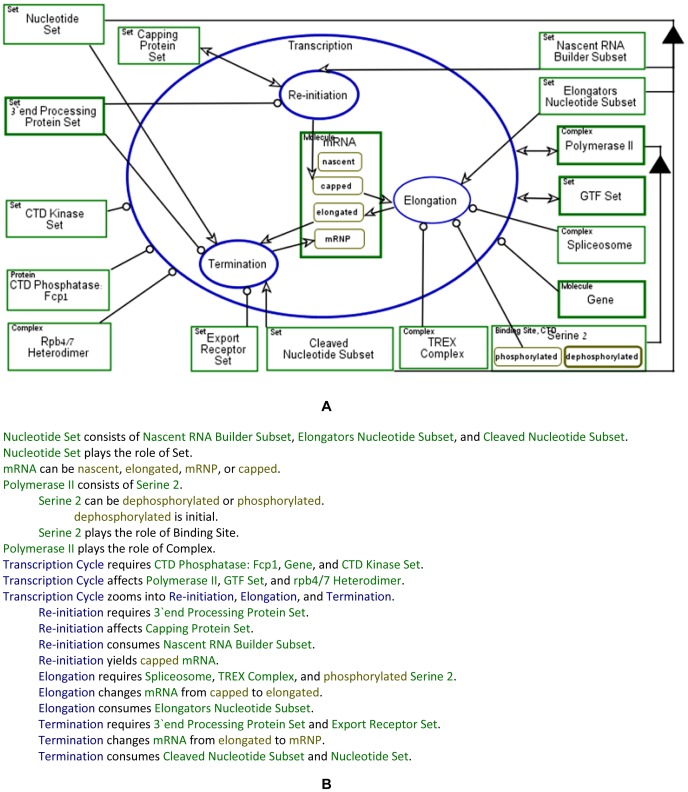
The transcription process bi-modal representation. (A) The Transcription process model. (B) The corresponding automatically-generated Object-Process Language (OPL).

In Figure 11A **Transcription Cycle** is zoomed into three subprocesses: **Re-initiation, Elongation** and **Termination**. The **mRNA** is created and modified during these **Transcription Cycle** subprocesses. The **mRNA** is created from a **Nucleotide Set**, which is consumed during **Termination**, as depicted by the consumption link, the arrow emanating from the **Nucleotide Set** object into **Termination**. The **mRNA** is created during the **Re-initiation** process in its **capped** state, as denoted by the state-specified result link from the **Re-initiation** process to the **capped** state of **mRNA**. During **Elongation**, **mRNA** is synthesized and processed, resulting in a state change from **capped** to **elongated**. During **Termination**, multiple proteins are recruited onto the **mRNA**, including **Export Receptor Set**, which is a set of factors that support export of **mRNA** with **Rpb4/7** into the cytoplasm, changing its state from **elongated** to **mRNP**. Figure 11B presents the corresponding Object-Process Language (OPL) text, which is a subset of natural English, generated automatically by OPCAT. OPL sentences concisely specify in text exactly what the Object-Process Diagrams (OPDs) express graphically, catering to people who are more inclined to comprehend complexities of systems by reading text (popularly referred to as “right-brain people” according to the theory of “left-brain or right-brain dominance”) rather than by diagrams (preferred by “left-brain people”).

OPL sentences specify (1) the structure of the system and (2) the behavior of the system, in particular how processes change object states, how they create new objects (molecules or complexes), how they consume existing ones, and what objects (called enablers in OPM) are required in order for a process to take place even though they are not affected. Two examples of structure sentences appearing in Figure 11B are: (1) “**Nucleotide Set** consists of **Nascent RNA Builder Subset, Elongators Nucleotide Subset**, and **Cleaved Nucleotide Subset**.” (2) “**Polymerase II** consists of **Serine 2**.”. Examples of behavior sentences are: (1) “**Termination** consumes **Nucleotide Set**.” – a consumption sentence, (2) “**Elongation** changes **mRNA** from **capped** to **elongated**.” – a state-change sentence, specifying the state before (**capped**) and after (**elongated**) the process **Elongation** took place, and (3) “**Elongation** requires **Spliceosome**, **TREX Complex**, and **phosphorylated Serine 2**.” – an enabling sentence, specifying the exact list of objects (molecules and/or complexes) required for the **Elongation** process to take place.

As these examples show, not only can the English-translated OPL sentences be understood easily by biologists who are not conceptual modeling experts; these sentences include unambiguous, essential information for understanding the structure, behaviour, and function of the biological system at the various levels of hierarchy. In contrast, text in research papers is written in free, unconstrained language. This freedom allows paper authors to write complicated sentences that on one hand are hard to follow, and on the other hand do not provide complete information, either because this information is assumed to be known, or because it is not known. Most often, neither the former nor the latter case are explicitly declared. In contrast, since OPL is derived automatically from a formal OPM model, which is guaranteed to be consistent, the text in each sentence expresses an unambiguous model fact that is based on the literature and/or new findings.

While modeling facts expressed in different research papers, contradictions may pop up. These are discovered while attempting to execute the unified model. Indeed, we have accidently encountered at least one case of such a contradiction between two published papers, which is beyond the scope of this paper. The likelihood of detecting such contradiction by merely reading free text of two different papers is very slim. This points out to another benefit of our model-based approach. Such contradictions, which will be reflected also in the OPL text, can be resolved by searching for supporting evidence in related papers or executing actual lab experiments to support one claim and refute the other. Once decided, the correct facts are incorporated into the graphical OPM model and they will be automatically reflected also in the text.

**Figure 12:**
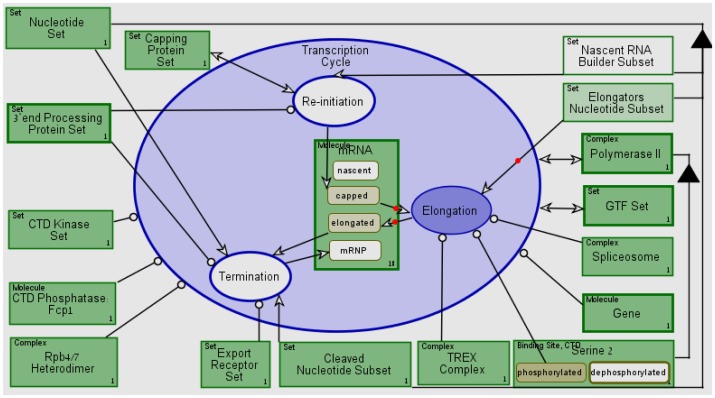
The execution of the transcription model. Here shown a snapshot of the **Elongation** process being executed (and therefore highlighted in purple), and the **mRNA** changes states from **capped** into **elongated**. See supplemental movie SV1 for **Re-initiation** process non-deterministic execution.

Figure 12 presents a screenshot of the **Transcription Cycle** process during its execution using OPCAT. Our conceptual model execution includes qualitative execution of a transcription cycle of a single, representative mRNA molecule. **Elongation** (colored in dark purple) is being executed, while the **Elongators Nucleotide Subset** object is being consumed, as denoted by the red dot along the consumption link, and **mRNA** changes states from **capped** to **elongated**. A transcription cycle execution record can be downloaded from [45] and for an explained partial execution see Video S1.

**Figure 13:**
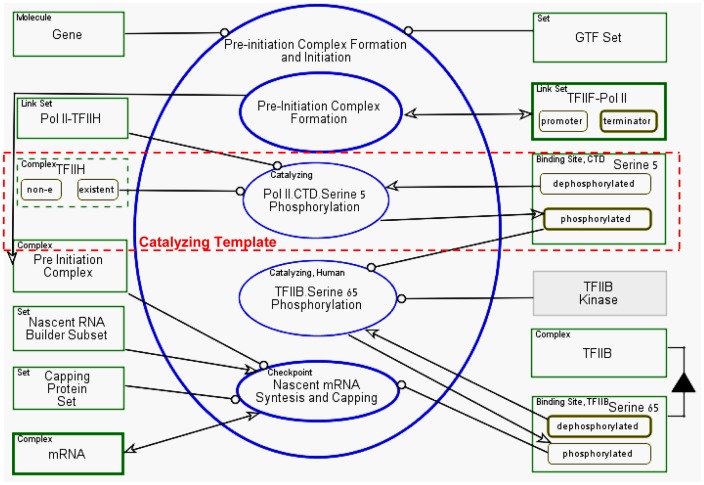
Pre-initiation complex formation and initiation model. In this example we apply the Catalyzing - Substrate Changed modeling template for modeling serine 5 phosphorylation (surrounded by dashed square) by TFIIH Kinase. TFIIB Kinase is still conjectured and therefore highlighted in grey.

The **Re-initiation** process is further zoomed (diagrams not shown), exposing two subprocesses: **Pre-initiation Complex Formation and Initiation** and **Promoter Clearance**. Figure 13 presents a diagram, in which the **Pre-initiation Complex Formation and Initiation** process is further in-zoomed. Its second and third subprocesses, **Pol II.CTD.Serine 5 Phosphorylation** and **TFIIB.Serine 65 Phosphorylation**, are not further in-zoomed, as depicted by their thin surrounding ellipse contour. Both are atomic phosphorylation functions, classified as *Catalyzing – Substrate Changed* molecular functions and modeled using the appropriate template, as highlighted in Figure 13 with a dashed line applied to **Pol II.CTD.Serine 5 Phosphorylation**, where TFIIH is the kinase.

**TFIIH** (see Figure 13) is defined as environmental object (surrounding line is dashed), meaning that its existence is randomly chosen during execution. If it is chosen to be **non-e** (non-existent), the **Pol II.CTD.Serine 5 Phosphorylation** and **TFIIB.Serine 65 Phosphorylation** processes will not be executed, resulting in abortive RNA. This flow of events exemplifies a possible non-deterministic execution of the model. Using environmental objects, the model shows not only a “successful” transcription process, but also includes the possible failure scenarios, such as abortive RNAs (See Video S1). We note that a successful transcription cycle is known to be a rare event [38], yet it is the only one leading to mRNA synthesis. Thus this transcription non-deterministic model may show the abnormal termination options, which is probably highly valuable for understanding the source of various defects and diseases.

#### 2.2 The utility of conceptual model-based systems biology

Our framework assumes the existence of a “ground truth” conceptual model: a model kernel in a specific molecular biology research area that was constructed manually based on the best available knowledge from the literature, validated by the best experts in this specific research area, and adjusted to execute correctly and fit the experimental data. Our Conceptual Model-based Systems Biology framework includes a set of methodological guidelines that help the biologist to (1) incorporate her or his findings into the existing model, thereby augmenting and evolving it, making sure it is still executable and consistent, (2) identify potential knowledge gaps within the augmented model, and (3) if a knowledge gap is discovered, generate one or more hypotheses, incorporate it into the model, and test the model before the design of another set of one or more lab experiments aimed to close this gap. The model with the conjectured hypothesis can be tested by comparing its execution to fit the experimental findings. If the ground truth model is augmented and no knowledge gap is discovered, the facts that have been added can potentially become part of the new, augmented ground truth model, and this is how the model evolves over time.

### 3. Detecting Knowledge Gaps and Model Errors

During the attempts to unify the data related to the mRNA transcription and decay processes, into one executable mechanistic OPM model, we have detected knowledge gaps and model errors of various types. Detecting a knowledge gap during manual OPM model construction regarding translation factors localization in P-bodies is described in [46], resulted in raising and experimentally proving a conjecture about eRF3 location in the P body. We note that in this work OPCAT execution capabilities were incomplete and were not used. Also the biological modeling templates were not defined.

We define a *knowledge gap* as lack of knowledge regarding a specific detail of some process and/or object in the system being modeled. We define a *model error* as an inconsistency regarding a specific detail of some process and/or object in the system being modeled. Knowledge gaps and model errors prevent a given system model from being able to completely and satisfactorily explain or execute the behavior of that system.

Model errors are detected automatically during model execution. Knowledge gaps can arise under the following possible circumstances: (1) *manually*, while trying to model some fact that is stated in the literature using a modeling template of one of the three molecular functions (see Figure 7, Figure 8 and Figure 9) or represent the temporal execution order of two or more processes, or (2) *automatically*, during model execution, as a result of detecting model errors in the model of the system under test. This model errors are raised when the model does not execute as the suggested mechanism or the execution outcomes does not match the expected experimental outcomes.

Our qualitative model execution (with one instance defined for each model entity) can help expose modeling errors resulting from temporal aspects, incorrect control flows, or wrong outcomes. The execution can detect, (1) object-related discrepancies, such as missing or redundant objects (e.g., association objects or some molecule), or (2) state-related discrepancies, such as incorrect state or an object being at more than one state at the same time. The detected errors results from process-related discrepancies, such as missing, temporally misplaced, or redundant molecular functions. After detected, the relevant process should be adjusted to enable successful model execution. Examples of *missing object* error and *incorrect state* error follow.

**Figure 14:**
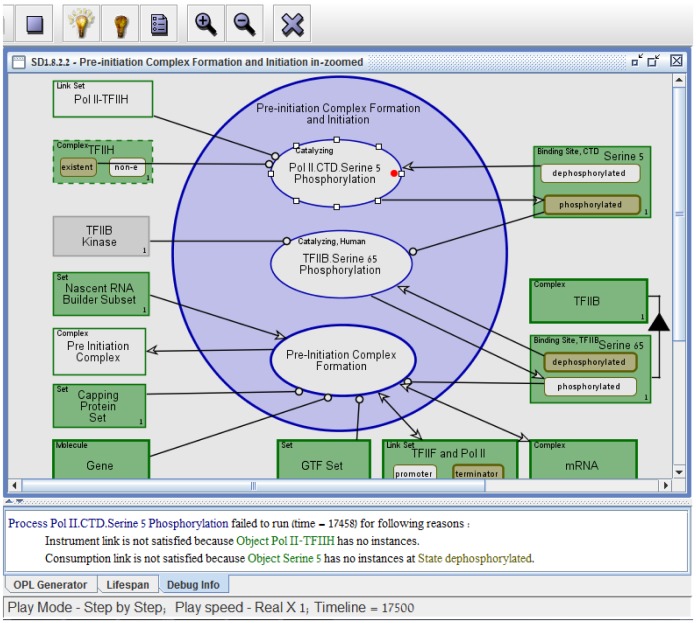
Example of two errors found during model execution. The transcription model execution halts during the **Pol II.CTD.Serine 5 Phosphorylation** process with errors presented in the lowest frame (see Video S2). The first error is a *missing object* error and the second is an *incorrect state* error.

While constructing and executing our transcription model, the control flow was found to be incorrect, indicating one or more modeling errors or gaps in our knowledge. Figure 14 is a screenshot of the model and this incorrect erroneous flow, causing halt of the system during execution. For example, the **Pol II.CTD.Serine 5 Phosphorylation** process halted execution with the following two errors indicated by our software:

“Process **Pol II.CTD.Serine 5 Phosphorylation** failed to run (time = 17458) for the following reasons:

1. Instrument link is not satisfied because object **Pol II-TFIIH** has no instances.
2. Consumption link is not satisfied because object **Serine 5** has no instances at state **dephosphorylate**”.

As exemplified in Figure 14, the precondition of the **Pol II.CTD.Serine5 Phosphorylation** process includes three instruments: (1) the existence of the object **TFIIH**, (2) existence of the object **Pol II-TFIIH** Link Set, i.e., recruitment of **TFIIH** to **Pol II**, and (3) the existence of the object **Serine 5** in its **dephosphorylated** state. The first error detected above is a *missing object* error since the **Pol II-TFIIH** object is non-existent (i.e., has no instances). It results from a temporal error, indicating that **TFIIH** was not recruited to **Pol II** prior to **Serine 5 Dephosphorylation**, as required. The second detected error is an *incorrect state* error since **Serine 5** is in the incorrect **phosphorylated** state. This execution error results from a missing molecular function. We indeed found that there is no specified molecular function that transforms **Serine 5** (located at position 5 of the C-terminal domain belonging to the Rpb1 subunit of the RNA Polymerase II) from its **dephosphorylated** state to its **phosphorylated** state.

As a result of detecting these errors, the model was corrected and then executed successfully. One of the corrected diagrams is in Figure 13, where the **Pol II.CTD.Serine 5 Phosphorylation** and **TFIIB.Serine 65 Phosphorylation** processes where changed to be executed after **Pre-Initiation Complex Formation** process (and TFIIH recruitment) and before **Nascent mRNA Synthesis and Capping**, since **TFIIB.Serine 65 Phosphorylation** is a condition for transcription initiation and capping [42].

The errors exemplified above are modeling errors, which are made often by the system modeler. Many such modeling errors were detected as a result of our model execution and fixed during the transcription model construction.

In addition to the modeling errors, which were fixed, we also detected 17 actual knowledge gaps, which are presented in Table 1. Knowledge gap number 11, which relates to the unknown dephosphorylation of TFIIB, was detected while cyclically executing the transcription re-initiation. This *incorrect state* model error was detected when the execution halted; indicating that the serine located at position 65 of TFIIB is not in the required dephosphorylated state. The other knowledge gaps were detected manually prior to model execution.

**Table 1:** Knowledge gaps found while modeling the mRNA transcription process.

	Knowledge Gap Type	Associated Molecular Function	Knowledge Gap
1	Unknown temporal order	Binding	When is Rpb4/7 recruited to RNA Polymerase II?
2	Unknown Object (binding molecule)	Binding	What molecule recruits Rpb4/7 to Polymerase II?
3	Unknown temporal order	Binding	When does Rpb4/7 bind FCP1?
4	Unknown temporal order	Binding	When does Rpb4/7 bind TFIIF?
5	Unknown temporal order	Binding	When does TFIIB bind FCP1?
6	Unknown temporal order	Binding	When does FCP1 bind TFIIF?
7	Unknown temporal order	Binding	What is the temporal dependency of Rpb4/7 recruitment and TFIIH and TFIIE recruitment to PIC?
8	Unknown temporal order	Binding	When does Pol II Bind TFIIF?
9	Unknown temporal order	Transporting	When does Pol II change location from terminator to promoter?
10	Unknown object (binding domain)	Binding	What domains of FCP1 does rpb4/7 bind to?
11	Unknown object (phosphatase)	Catalyzing	What molecule dephosphorylates TFIIB serine 65?
12	Unknown molecular function	Missing Molecular Function	How is Fcp1 inhibited?
13	Unknown object	Binding	What molecule binds Fcp1 to inhibit its activity?
14	Unknown object (kinase)	Catalyzing	What is the Ser7 kinase?
15	Unknown object	Binding	What molecule recruits Ser7 kinase?
16	Unknown object (phosphatase)	Catalyzing	What is the Ser7 phosphatase?
17	Unknown object (binding molecule)	Binding	What molecule recruits Ser7 phosphatase?

Our ability to detect most of the actual knowledge gaps manually may be due to the fact that many of the modeled mechanisms were completely unknown, making it hard to construct the model initially. Another reason can be that the model is medium sized and not overly complex. However, we expect that in larger, more complex models, concrete knowledge gaps will be more difficult to detect by humans static inspection, yet they can be detected automatically by trying to execute the model in the same manner exemplified above.

We note that knowledge gap can also result from an inconsistency between two or more temporal facts stated in two or more different research papers. This might indicate an incorrect interpretation of the experimental results in one of the research papers between which a contradiction has been detected through the model. We have indeed found a discrepancy between findings stated in two papers in the decay part of our larger mRNA lifecycle model (not presented here).

After the model was constructed and evaluated to be consistent, one can replace the execution mode from the “halt execution” mode to the “skip process” mode. In the “halt execution” mode, instrument links are used, representing a precondition that must hold for the model to continue its execution. In the “skip process” mode, condition links are used, providing for skipping a process whose precondition is not met. In the “skip process” mode, unsatisfied conditions do not halt the model, but rather skip the process and continue executing. This enables analysis of system perturbations, such as mutations, and execution of non-deterministic models (see Video S1).

#### 3.1 Knowledge gaps classification

Having modeled the mRNA transcription cycle as well as the mRNA decay process (which is not presented in this work), we fixed the modeling errors, highlighted the actual knowledge gaps, and analyzed their characteristics. Based on this analysis, we propose a classification of knowledge gaps that might arise as a result of qualitative conceptual modeling of molecular biology system mechanisms.

Our knowledge gaps classification is based on the three molecular functions—catalyzing, binding/dissociating, and transporting—and their modeling templates. Knowledge gaps might stem from (1) lack of knowledge regarding a molecular function at some point in the model, (2) the completeness of the molecular function template and the structure of the participating objects in the template (e.g., missing knowledge on binding sites or enzymes), or (3) the temporal execution order of a molecular function within the scope of its higher level biological process. Accordingly, we classify knowledge gaps into the following three types.

**Unknown Molecular Function** – Lack of knowledge about whether a molecular function F happens in a certain place under certain circumstances. For example, it has been sown that TFIIB inhibits the phosphatase activity of FCP1 [47]. When we tried to incorporate this finding into the transcription model as a molecular function, knowledge gaps emerged, preventing straightforward modeling of this assertion. We can assume that the inhibition is due to binding of some unknown molecule A to Fcp1. This is represented in the model using the unknown *Binding* molecular function between Fcp1 and some unknown molecule A. Consequently, the CTD de-phosphorylation function is inhibited, because Fcp1 is not free to carry out its “usual” activity due to its binding to A. The knowledge gap here is weather a *Binding* molecular function between A and Fcp1 occurs (see Table 1, row no. 12).

**Unknown Object** –Lack of knowledge about an object (such as a molecule) that participates in a molecular function. For example, while it is obvious that the molecular function of TFIIB.serine 65 dephosphorylation is needed for executing a temporally coherent model, the details of this molecular function are unknown (see Table 1, row no. 11). The unknown identity of the TFIIB Kinase is highlighted in grey in Figure 13. Another example is a knowledge gap regarding the identity of A in the case of inhibiting the phosphatase activity of FCP1, by binding [47]. We might conjecture that it is TFIIB, as this is in line with the fact that TFIIB binds fcp1 [44]. However, this is a mere conjecture that must be proved empirically.

**Unknown Temporal Order** – Lack of knowledge about the temporal order of the molecular function along the model timeline. It is unknown whether a molecular function F, which is known to happen, must happen before or after another molecular function F′, or whether F and F′ are dependent on each other and therefore must happen in parallel, or whether they are independent and therefore each one of them can happen before, during, or after the other. An example of a temporal order knowledge gap is the unknown temporal order of the RNA Polymerase II to Rpb4/7 binding function: It is unknown whether it occurs during transcription termination, during transcription initiation, or between these two processes (see Table 1, row no. 1).

Table 1 summarizes the 17 knowledge gaps we found in the transcription model. Of those, all but knowledge gap number 11 were found manually, while constructing the model. Each knowledge gap is phrased as a question, and for each one, the associated molecular function and knowledge gap type are recorded.

After a knowledge gap has been detected, be it manually or automatically, the model needs to be augmented with a conjecture that enables its execution. These conjectures, highlighted in grey in the model (such as TFIIB Kinase in Figure 13), are verified by model execution and then must be verified empirically. If there is more than one alternative conjecture, experimental results will determine which one is correct, so the model can be updated accordingly, serving as an evolving reliable knowledge resource.

We have also worked on a larger model, the mRNA decay model, which is not presented in this paper. The mRNA decay model comprises 130 objects and 65 processes, of which 41 are leaf, atomic processes, and 24 are higher level. In this mRNA decay model, which runs 9 in-zooming levels deep, we found 24 knowledge gaps, of which 13 were related to *unknown temporal orders* and 6 were *unknown objects*. Like in the mRNA transcription model which is the focus of this paper, only one knowledge gap was of the type *unknown molecular function*. Most of the knowledge gaps in both models were related to (1) *unknown temporal orders*: 47% and 50% in the transcription and mRNA decay models, respectively, and (2) *unknown objects*: 47% and 25% in the transcription and mRNA decay models, respectively.

Interestingly, since the mRNA decay is a newer, more cutting-edge research subject, experimental results that were related to completely unknown mRNA decay mechanisms gave rise to four wider knowledge gaps of a new kind, which we call *unknown mechanism*. Each unknown mechanism involves a set of several unknown molecular functions. Thus, a hierarchy of knowledge gap types can be defined, in which *unknown mechanism* is the widest, followed by *unknown molecular function*, *unknown object* and *unknown temporal order*.

## Summary and Discussion

We have proposed a Conceptual Model-based Systems Biology framework. Our framework enables multi-layer qualitative modeling and model execution, as well as model-based elicitation and classification of knowledge gaps in molecular biology systems. We also show how model execution detects model errors and enhances the construction of a mechanistically coherent model.

The framework adapts Object-Process Methodology (OPM) to the domain of systems biology. OPM fits the task at hand as it enables concurrent representation of the system's structure—the objects that comprise the system, and its behavior—how processes transform objects over time. OPM is a conceptually rich, graphical language which has the capacity to capture the variety of biological information by connecting stateful objects (i.e., molecules) to biological processes that transform them: create or destroy them, or change their states at various levels of detail.

Modeling the mRNA transcription cycle as a case in point, we started with this high level cell function and modeled increasingly detailed processes, along with the objects participating in these processes. This case study has demonstrated modeling of molecular processes, such as complex formation, localization and trafficking, molecular binding, enzymatic stimulation, and environmental intervention. While this paper has focused on the mRNA transcription case study, using OPM for conceptual modeling in systems biology is by no means limited to this particular subsystem. Indeed, we have been applying OPM to model the mRNA decay process, which is the focus of current work in progress, and the Glycolysis metabolic pathway, part of which we present in Figure S1. Similar to the Gene Ontology (GO) definitions, at the lowest level of our framework, all biological processes boil down to three basic molecular functions: catalysis, binding/dissociation, and transporting. The simultaneous representation of structure and behavior via objects and processes, along with the modeling templates, provide for the ability to focus on particular molecules of interests and follow their changing role over time in complex biological processes. The ability to follow molecules as they participate in multiple processes can help discover multi-functional molecules, such as Rpb4/7, which has a key role in each major stage of the mRNA lifecycle [1], a finding that is emerging as a key feature of biological systems.

During modeling and execution of the mRNA transcription model, we discovered modeling errors and knowledge gaps. Our model execution can help expose modeling errors and knowledge gaps resulting from incorrect control flows or wrong execution outcomes. The execution can detect, (1) object-related discrepancies, such as missing or redundant objects (e.g., association objects or some molecule), or (2) state-related discrepancies, such as incorrect state or an object being at more than one state at the same time. Many model errors were discovered during model construction and execution, and the model was adjusted accordingly in an iterative improvement process, until we were satisfied with its execution flow and its agreement with published results, weeding out false positives as much as we could and leaving only “true” knowledge gaps.

Identification and classification of knowledge gaps is a valuable feature of the framework, as it suggests where research should focus and whether conjectures about uncertain mechanisms fit into the already verified evolving model. From a quantitative viewpoint, our mRNA transcription model includes 50 objects and 37 processes, 24 of which are low-level processes. In this model, we detected 17 actual knowledge gaps. These were related to molecular functions and classified into three types: unknown molecular function, unknown object, and unknown temporal order. About half of the knowledge gaps (eight of 17) related to temporal aspects (*unknown temporal order* type), another eight were unknown biological objects participating in some molecular function (*unknown object* type), and one related to an unknown molecular function (*unknown molecular function* type). We also demonstrated how our executable framework is capable of detecting temporal gaps and unknown molecular functions in a straightforward manner. In a more complex mRNA decay subsystem, which we also modeled, most of the knowledge gaps were also *unknown temporal order* and *unknown object* types. In the mRNA decay model we found a fourth, wider knowledge gap type—*unknown mechanism*, which comprises several unknown molecular functions, giving rise to a hierarchy of knowledge gap types.

Knowledge gaps can emerge from model execution using other conceptual formalisms. For example, temporal inconsistencies were reported when comparing *Caenorhabditis elegans* vulval development model execution with experimental results, using the Statecharts qualitative method [17]. Moreover, some of the questions and knowledge gaps might have been exposed by examining known facts without constructing the model and executing it. Yet, a systematic approach enforced by the modeling activity and the model execution may greatly enhance the detection of inconsistencies and the elicitation of knowledge gaps. Moreover, the model may also serve as a vehicle to resolve the detected inconsistencies and test conjectures related to knowledge gap resolutions.

The model can provide a top-level holistic functional view, such as gene expression, and gradually expose details of both biological processes and the involved structures all the way down to such minute details as whether a given amino acid is phosphorylated. OPM's in-zooming/out-zooming capability enables gradual exposure of system details. By traversing across detail levels, this refinement-abstraction mechanism facilitates focusing on fine details of a particular subsystem via in-zooming, and getting an overall system view via out-zooming. For example, using the OPM modeling tool OPCAT and its query capabilities, we can inspect for each molecule of interest the flow of processes it undergoes and how each process affects it. It is this ability to have a holistic system view on one hand and to inspect low-level details on the other hand that researchers, immersed in an ocean of details, often miss.

The benefits of using our framework to a biology researcher also include the ability to coherently preserve, manage, and evolve knowledge about a system under study. Our framework captures and explicitly represents both established and conjectured qualitative mechanistic knowledge about the function, behavior, and structure of the systems at a wide spectrum of detail levels. The model is the means to relate disparate pieces of information into a comprehensive, system-wide conceptual framework, in which knowledge is arranged in a consistent hierarchical way. The sources of the knowledge pieces can be a result of one's experiments combined with facts known from the literature. Being formal, the model can be executed in a straightforward manner using model checking techniques [29] from Computer Science. An important outcome of this knowledge formalized organization is the ability to construct a widely-expressive mechanistic coherent model and expose knowledge gaps that can provide a basis for designing and executing experiments.

A main drawback of executable methods is their closeness to computational semantics [2], such as being based solely on object states or events, and lack of adequate abstractions needed for closing the gap between these basic computation-oriented concepts and the rich set of concepts needed for representing biological systems. As we have shown, OPM does enable the representation of a rich set of biological structures and behaviors. One drawback of an expressive conceptual language is the need to use a larger set of concepts and symbols than used in other modeling methods, though in OPM the size of this set is kept to a minimum: stateful objects and processes that transform them as entities, and several link types to express structure and behavior connections in a single diagram type.

Conceptual qualitative models are key for understanding the system's underlying mechanisms, which are a result of the quantitative findings. Indeed, the model that we have developed so far is qualitative in nature; it does not represent continuously changing compartmental concentrations of reactants or stochastic data that are required to formalize quantitative models. Since our model is qualitative in nature, we map each experimental quantitative outcome to be incorporated into the model as Boolean. For example, 70% deactivation of some process is mapped in our model as 100% deactivation of that process. Yet, our approach is capable of modeling kinetic coefficients of reactants by using multiple instances of the biological objects, as exemplified in the model of the Glycolysis pathway (see Figure S1).

The proposed framework can help conceptualize an incomplete complex molecular biology system, drive execution, and support hypothesis generation and validation. After the model is constructed and evaluated through execution to match the known experimental data, it can be used to check hypotheses and to generate new ones. This can be done by perturbing the model or by incorporating into the model new hypothesized mechanisms and then matching its outcomes to known wet-lab experimental findings. If the new model with the conjectured mechanism yields the expected results, the conjecture is said to be consistent with the model and can be further tested experimentally. Our model may detect errors in biological mechanistic conjectures before conducting wet lab experiments. A restriction of the approach is that the model might yield false positives, i.e., indicate that an erroneous mechanistic conjecture is correct, because for lack of knowledge it executes correctly. Hence, model-validated conjectures still need to be confirmed via wet-lab experiments. On the positive side, though, many such experiments can be avoided or refined if the model proves them wrong in the first place.

A unique advantage of OPM is its bimodal representation: the graphic model is translated on the fly to Object-Process Language (OPL)—a subset of natural English that enables comprehension of the model by biologists who have no knowledge of the graphic symbols of OPM. The opposite translation of text to graphics is also possible. In the long run, we aim to automate the conceptual modeling task by targeted processing and analysis of natural language text from pertinent scientific articles. To start the process, a manually-constructed and conceptual ground-truth model of the kernel of the system under investigation must be developed and verified by human experts and via execution. This ground truth model will be the starting point for the automated model construction from literature text. In parallel, based on this work, we are also developing an automatic model verification framework [29].

## Materials and Methods

To create and execute the model, we used OPCAT [28]. We started by modeling established knowledge concerning the mRNA transcription re-initiation cycle from pertinent research papers. The list of facts and their references is presented in Table S2. Each basic OPM molecular function or molecular structure was defined with the relevant modeling template using the “role” feature in OPCAT. For the sake of executing the model, we defined one instance for each object class. We used the instrument links for defining process precondition in the “halt execution” mode, which was used for checking the model's consistency and detecting model errors. In this mode the system halts at any process whose precondition is not satisfied. Whenever the systems halted, we analyzed the detected errors and corrected them repeatedly, until the model execution terminated successfully. After the model was made coherent and executed as expected, the system may be converted to the “skip process” mode by changing the instrument links into condition links. During model construction and execution we discovered the knowledge gaps, recorded them, and classified them. For relevant knowledge gaps we added to the model the missing details as conjectures.

## Supporting Information

Figure S1 Diagram of the Glycolysis metabolic process.
(DOCX)

Table S1 Main OPM elements (as used in this work), with their symbols, definitions and execution semantics.
(DOCX)

Table S2 Transcription model facts and relevant references.
(DOCX)

Video S1 Record of a non-deterministic transcription re-initiation process execution.
(7Z) Click here for additional data file.

Video S2 Record of the transcription cycle model execution with model errors detected.
(7Z) Click here for additional data file.
