# Supplementary material for: Conceptual Model-Based Systems Biology: Mapping Knowledge and Discovering Gaps in the mRNA Transcription Cycle
Source: PLoS One. 2012 Dec 20;7(12):e51430. doi: 10.1371/journal.pone.0051430 (PMC3536069; doi:10.1371/journal.pone.0051430)
Supplement: Figure S1 — Diagram of the Glycolysis metabolic process. (DOCX) [file pone.0051430.s001.docx]

Supplemental Figure S1 for

Model-Based Systems Biology:

Mapping Knowledge and Discovering Gaps in the mRNA Transcription Cycle

Judith Somekh^a*^, Mordechai Choder^c^, and Dov Dori^a, b^

^a^Faculty of Industrial Engineering and Management, Technion, Israel Institute of Technology, Haifa 32000, Israel

^b^Engineering Systems Division, Massachusetts Institute of Technology, Cambridge, MA, USA

^c^Faculty of Medicine, Technion, Israel Institute of Technology, Haifa 32000, Israel


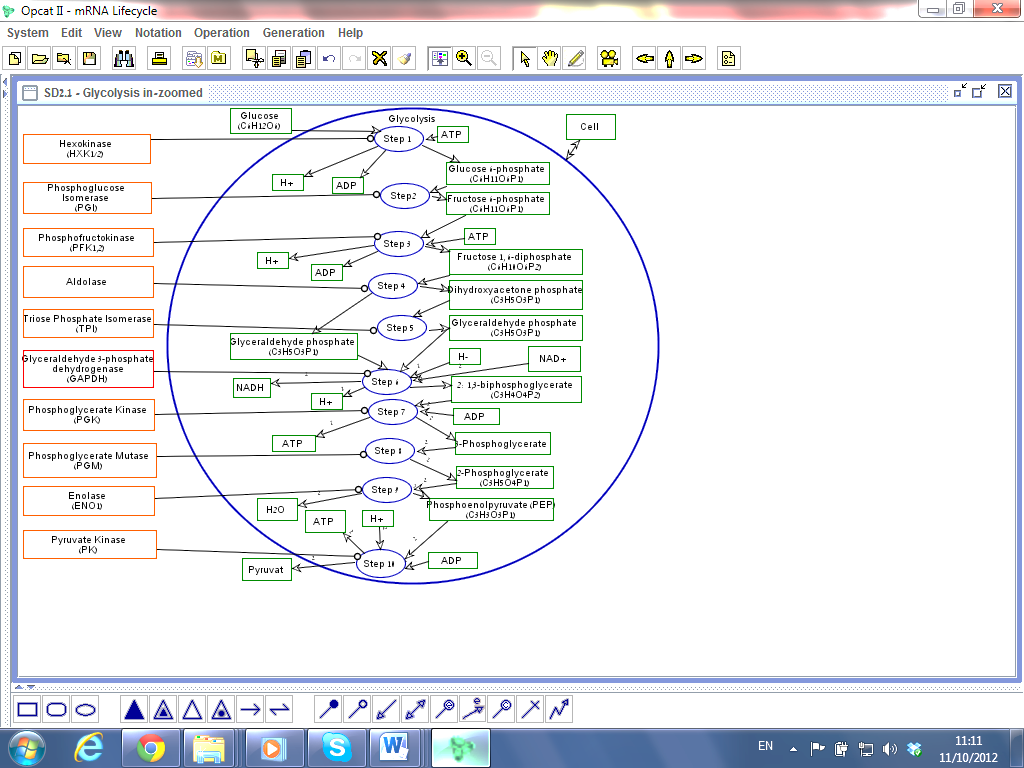


Figure S1. Diagram of the Glycolysis metabolic process. The multiplicity (kinetic coefficient) of consumed and created biological objects is depicted on the relevant arrows. For example, process named Step 7 consumes two molecules of ADP.
