# Supplementary material for: Conceptual Model-Based Systems Biology: Mapping Knowledge and Discovering Gaps in the mRNA Transcription Cycle
Source: PLoS One. 2012 Dec 20;7(12):e51430. doi: 10.1371/journal.pone.0051430 (PMC3536069; doi:10.1371/journal.pone.0051430)
Supplement: Table S1 — Main OPM elements (as used in this work), with their symbols, definitions and execution semantics. (DOCX) [file pone.0051430.s002.docx]

Supplemental Table S1 for

Model-Based Systems Biology:

Mapping Knowledge and Discovering Gaps in the mRNA Transcription Cycle

Judith Somekh^a*^, Mordechai Choder^c^, and Dov Dori^a, b^

^a^Faculty of Industrial Engineering and Management, Technion, Israel Institute of Technology, Haifa 32000, Israel

^b^Engineering Systems Division, Massachusetts Institute of Technology, Cambridge, MA, USA

^c^Faculty of Medicine, Technion, Israel Institute of Technology, Haifa 32000, Israel

Table S1. Main OPM elements (as used in this work), with their symbols, definitions and execution semantics

| Element | Concept Name | Symbol | Execution Semantics |
| --- | --- | --- | --- |
| Object | Systemic object | 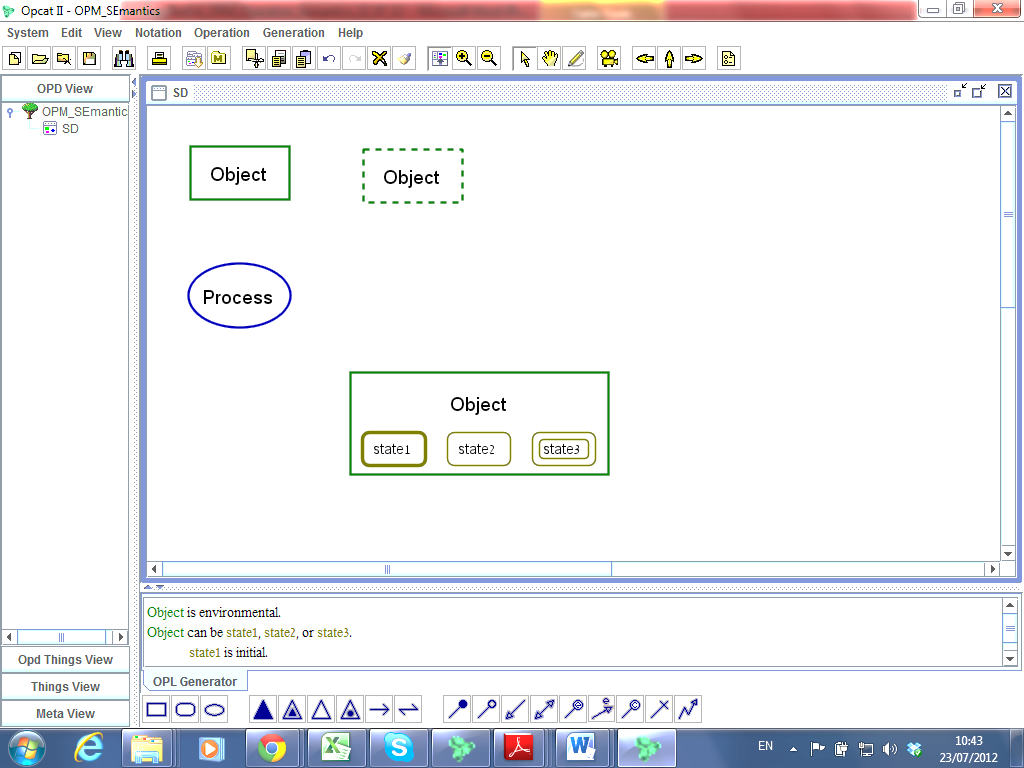 | An object which consist of a matter or a piece of information |
| Object | Environmental object | 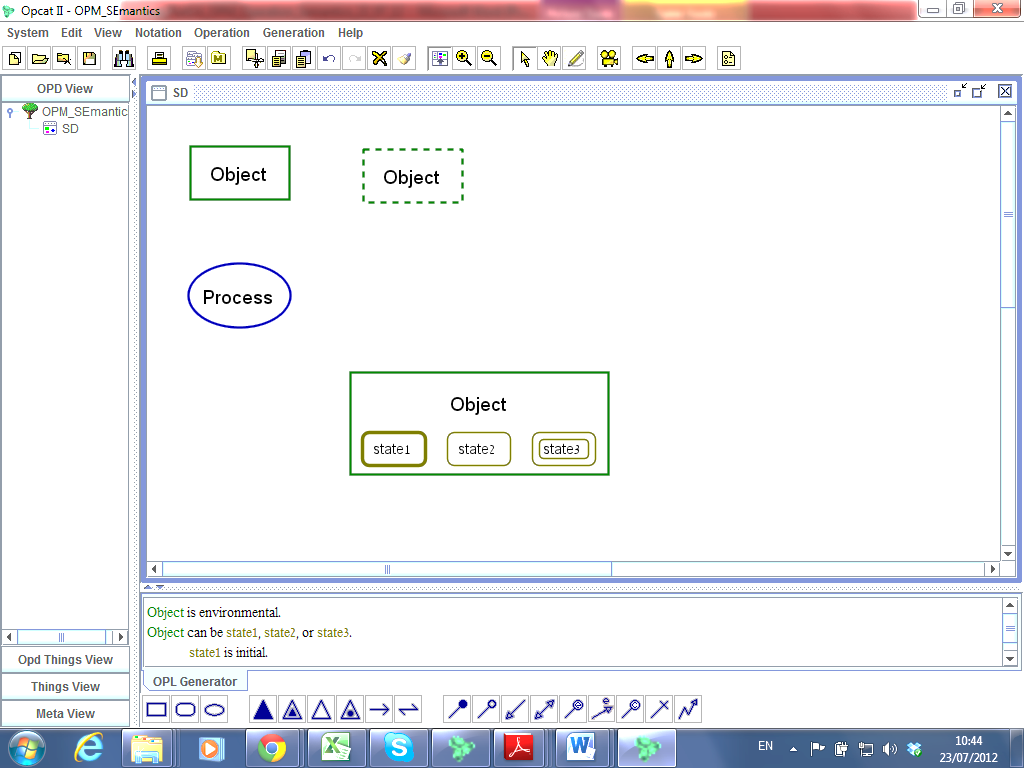 | An object which is external to the system, randomly generated |
| Process | Process | 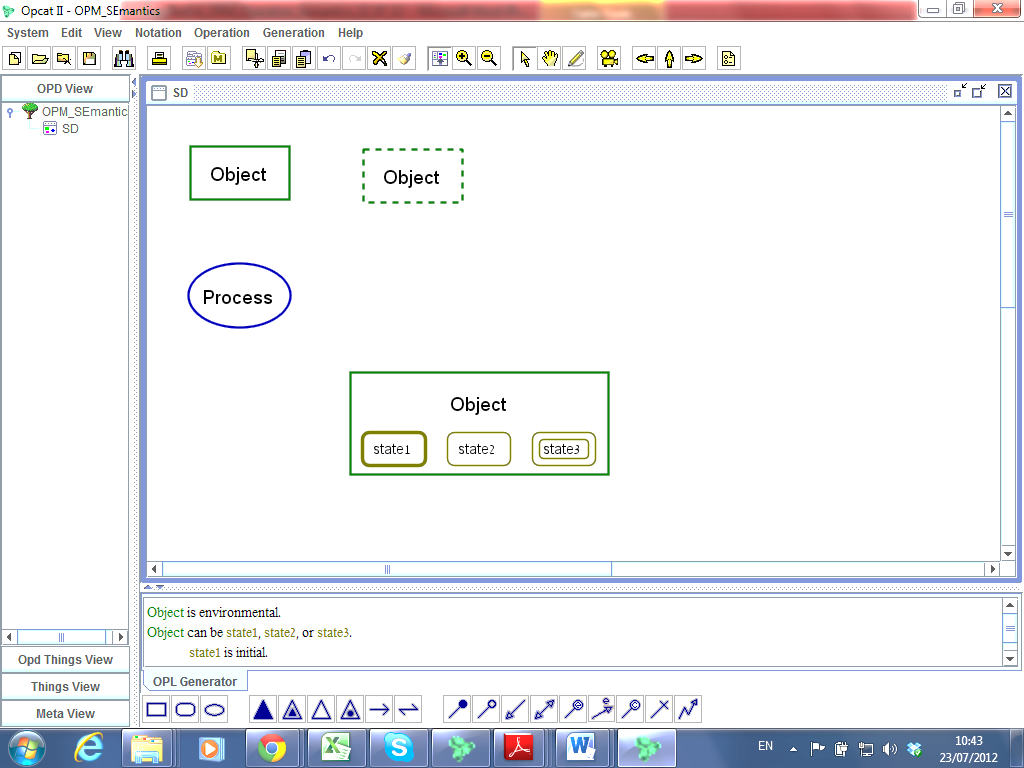 | A pattern of transformation that objects undergo |
| State | Initial/  Regular/  Final state | 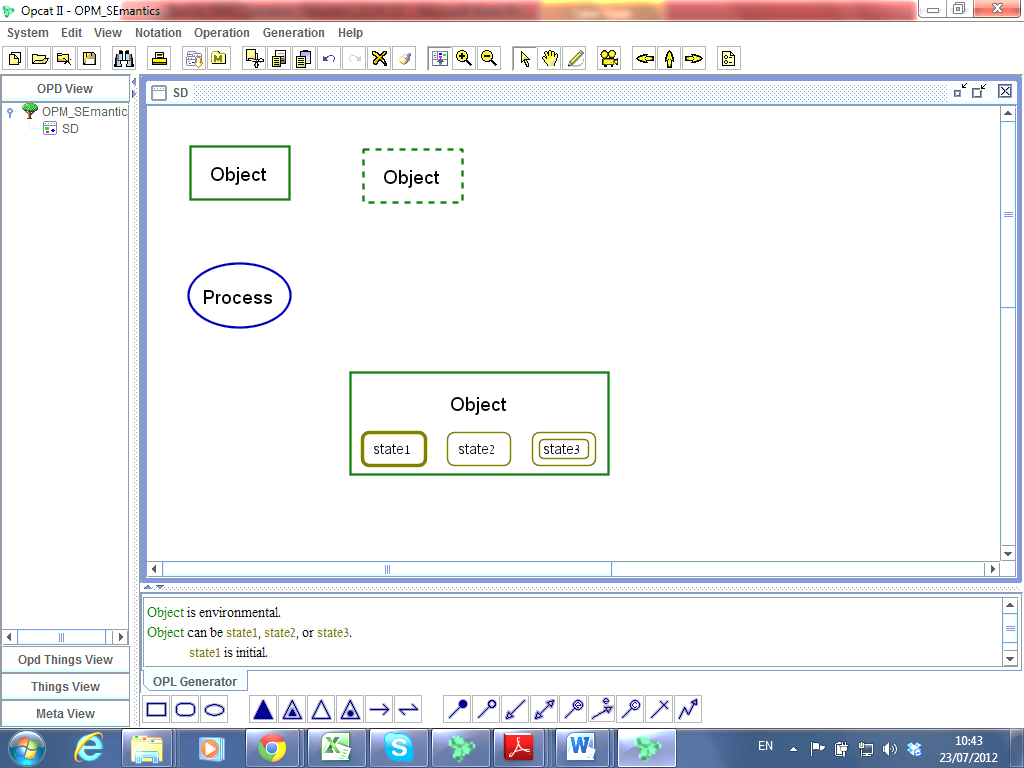 | An **initial** (state 1)/**regular** (state 2)/**final** (state 3) situation at which an object can exist for a period of time |
| **Object-Process Procedural Links:** Links representing Process Pre/Postcondition Object Set | | | |
| PreCondition Link | Condition link | 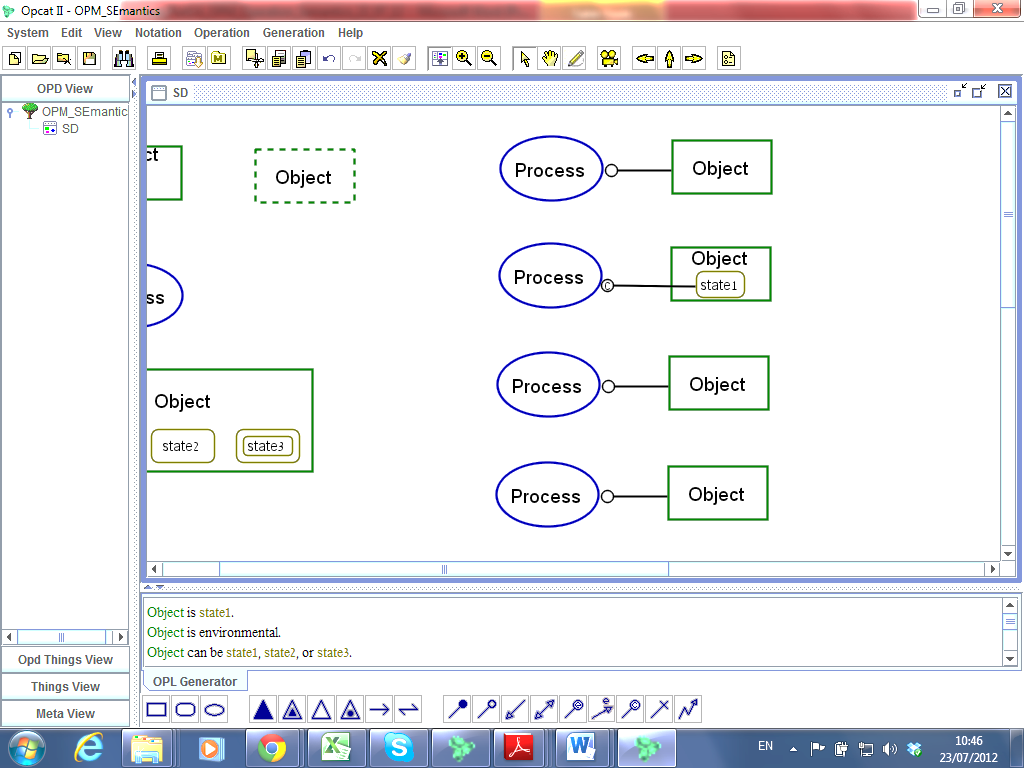  c  (A)  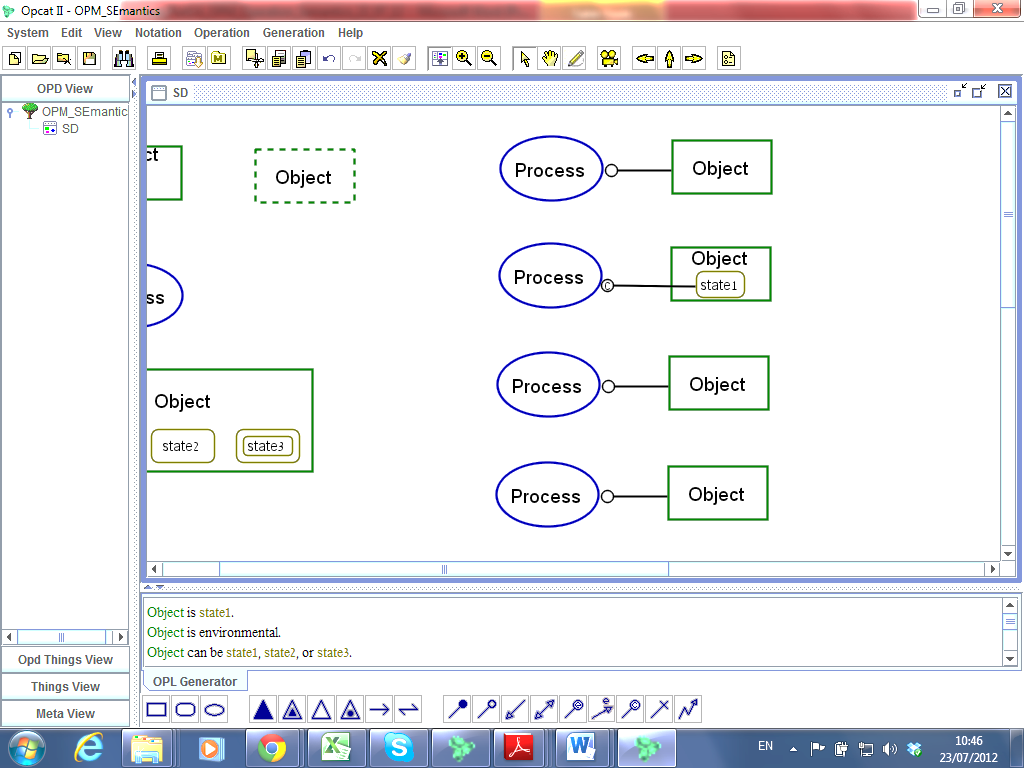  (B) | A link denoting a condition required for a process execution. The condition can be the existence of an object (A) or the existence of an object in some state (B). The condition is checked when the process is triggered. If the condition does not hold, the process is skipped and the next process (if any) tries to execute. |
| Precondition Link | Instrument link | 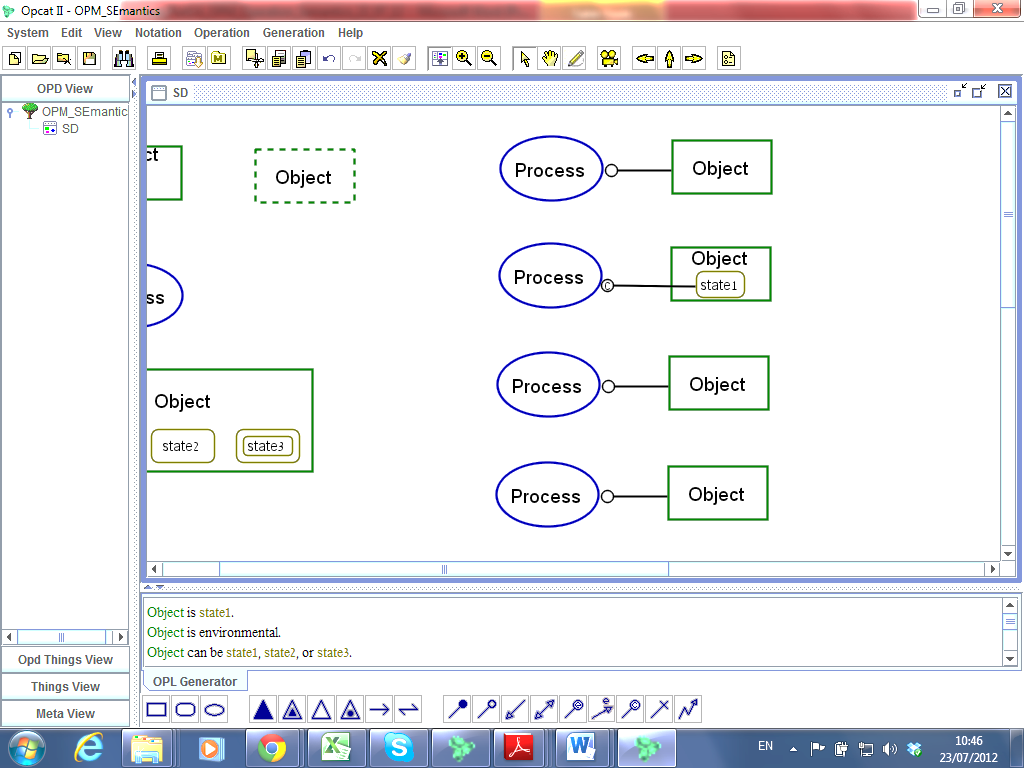  (A)  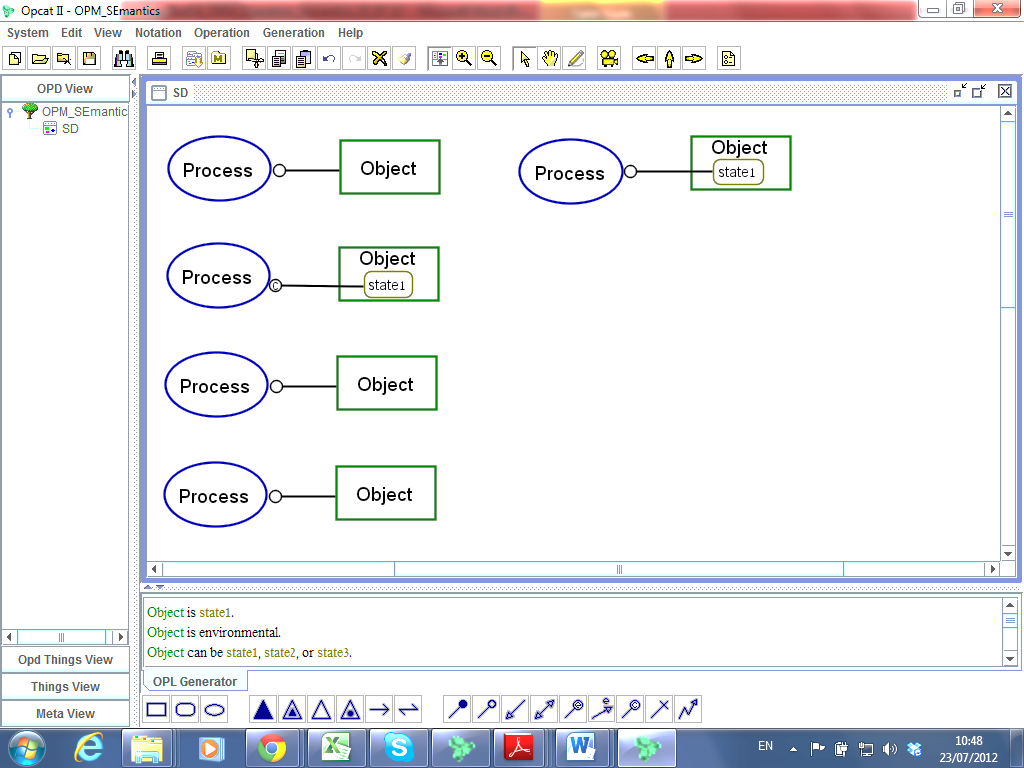  (B) | A link denoting a condition required for a process execution. The condition can be the existence of an object (A) or the existence of an object in some state (B). The condition is checked when the process is triggered. If the condition does not hold, the process waits for its availability, and the systems halts. |
| Precondition Link, Post-condition link | Consumption link | 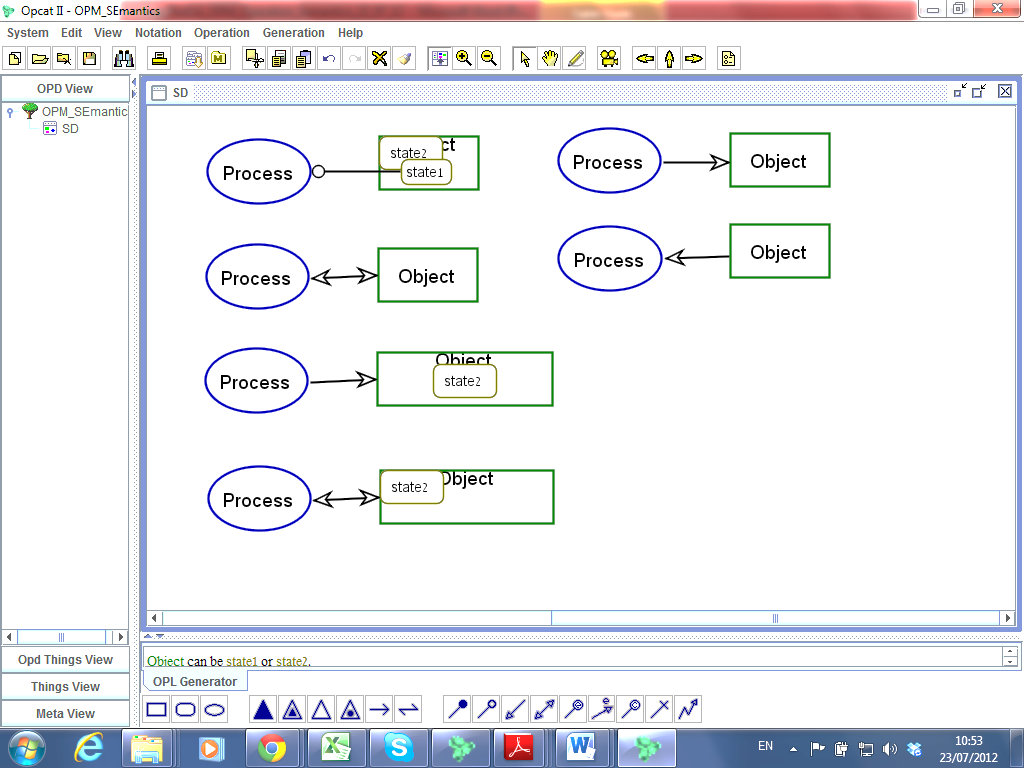  (A)  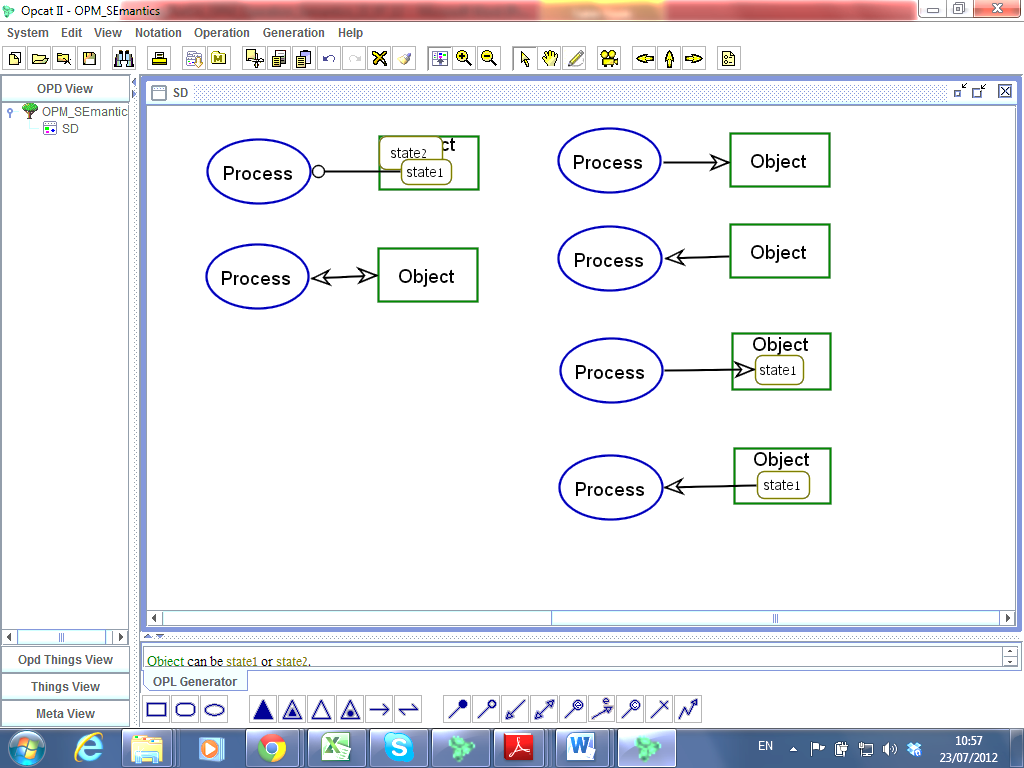  (B) | A link denoting that a process consumes an object (A) or an object at some state (B). The object (A) or object's state (B) existence is a precondition for process execution. |
| Post-condition link | Creation(Result) link | 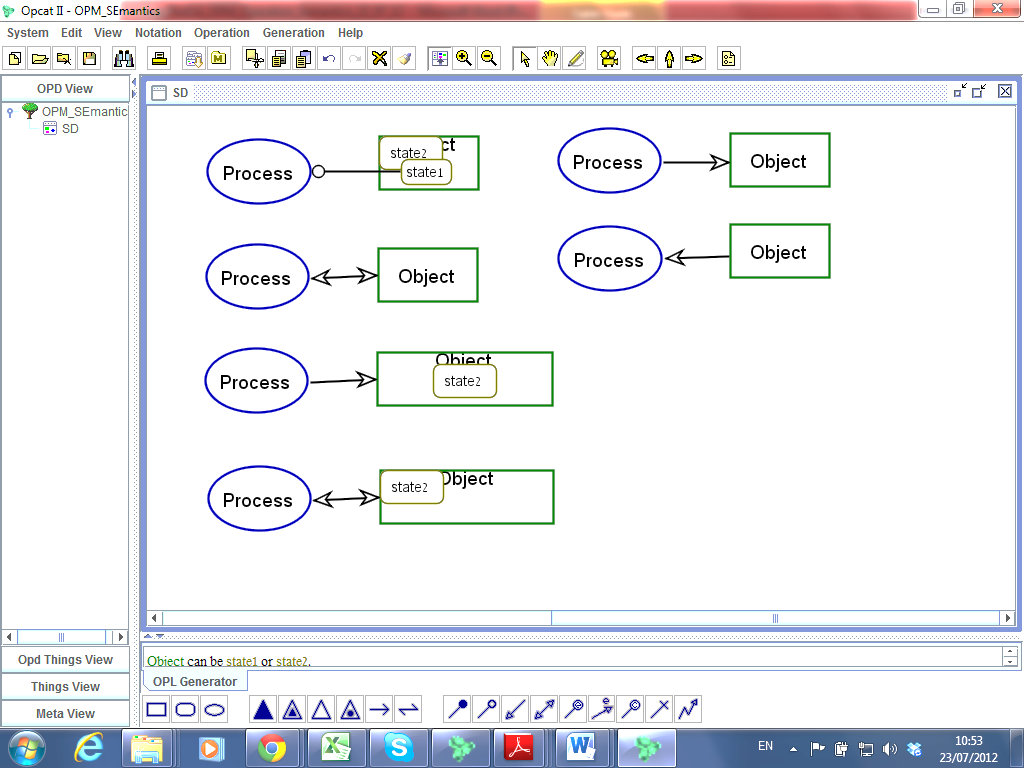  (A)  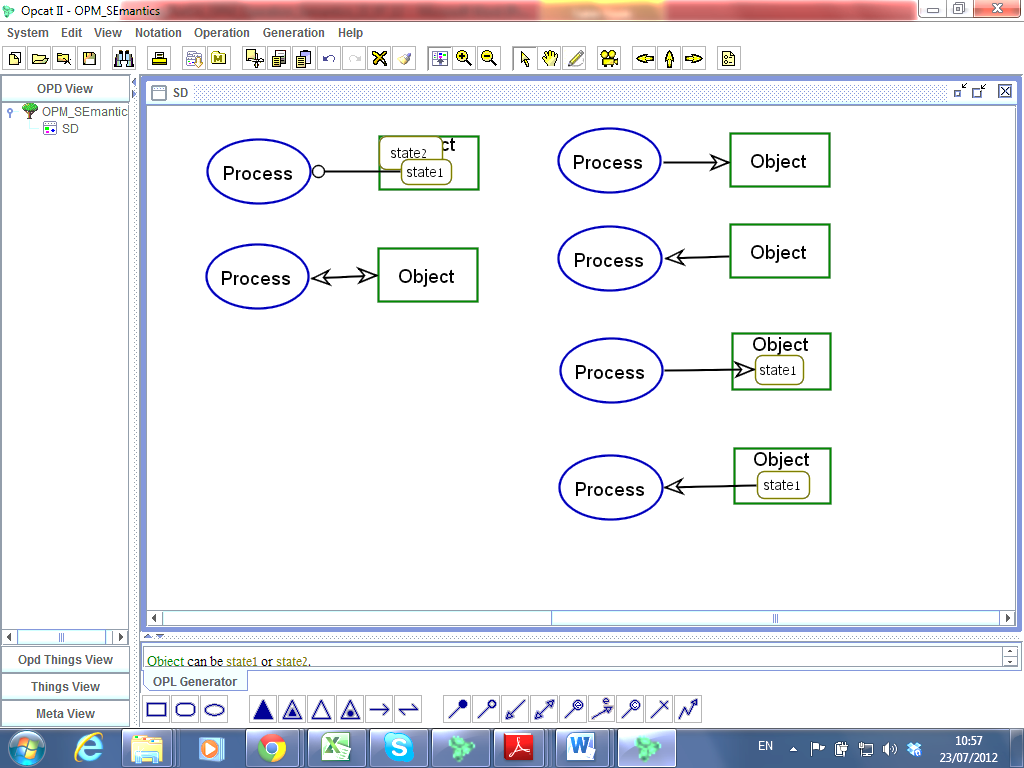  (B) | A link denoting that a process creates an object (A) or an object at some state (B). |
| Precondition Link, Post-condition link | Effect link | 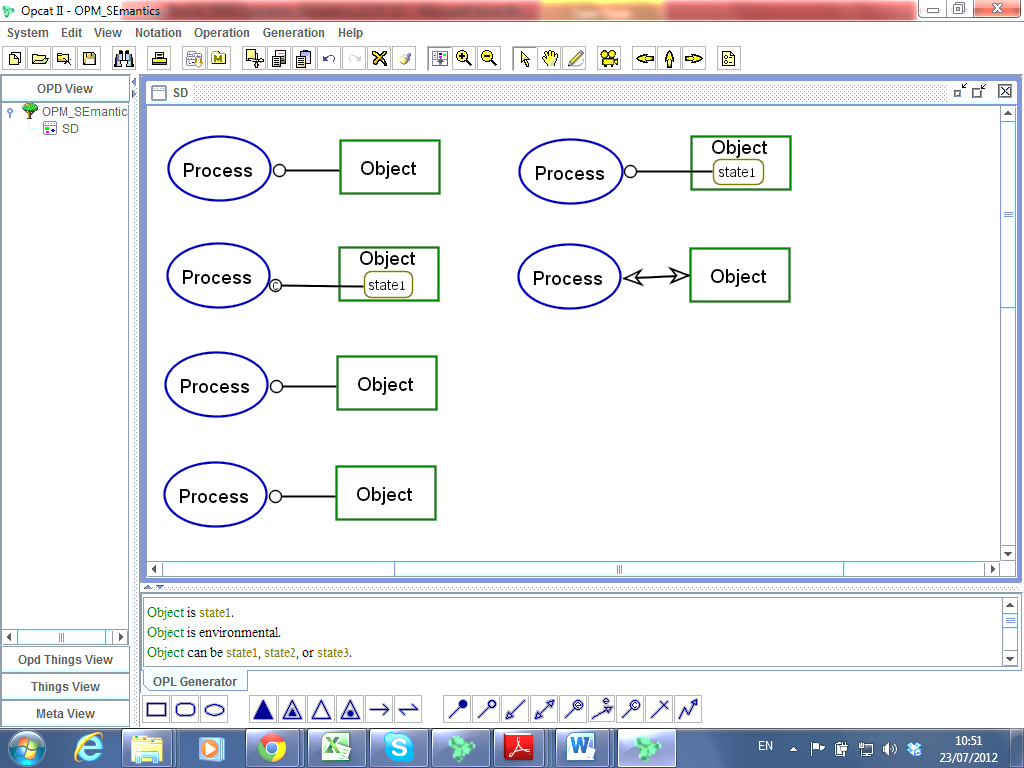 | A link denoting that a process changes an object. |
| Precondition Link, Post-condition link | Changing object state links (input and output links) | 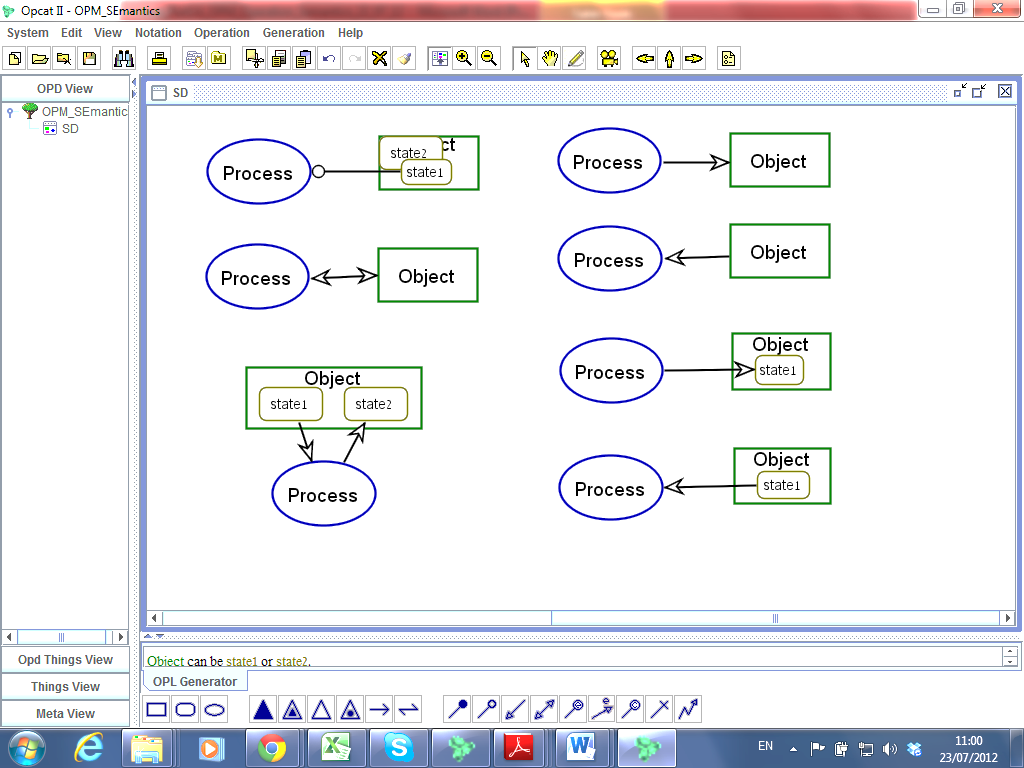 | Links denoting that a process changes an object from state 1 into state 2. The input link consumes state 1 and the output link creates state 2. Used to show the details of effect link, when a process is in-zoomed. |
| **Process-Process Links** | | | |
| Timeline changing link | Invocation link | 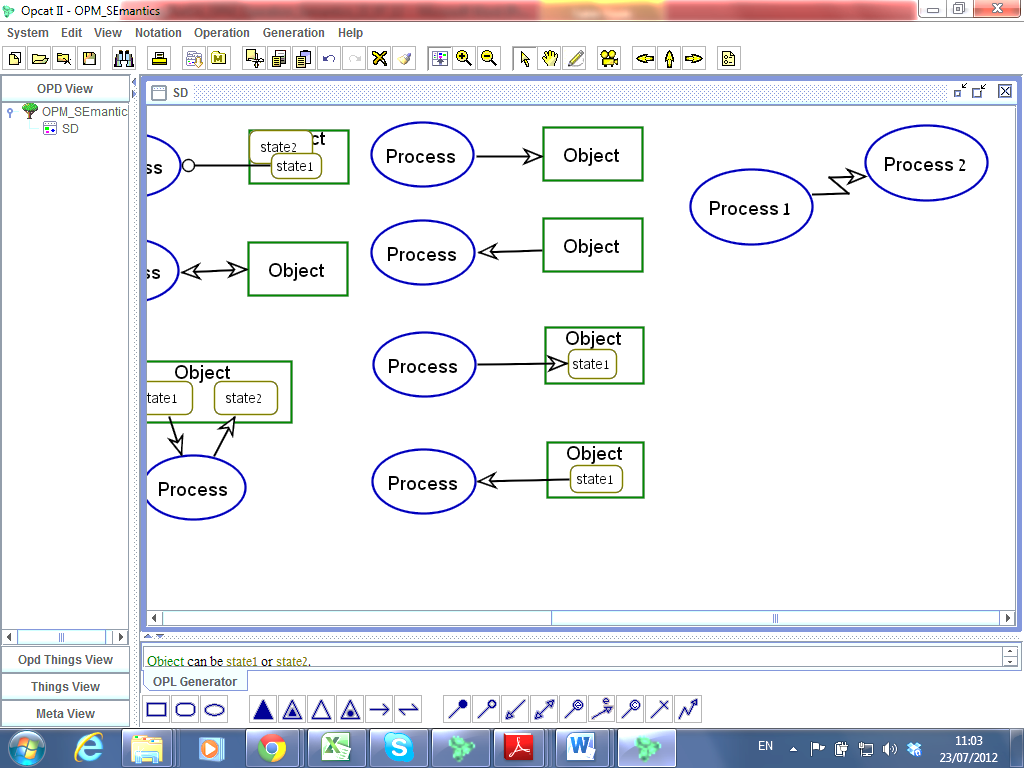 | A link denoting that a process, process 1, triggers (invokes) another process, process 2, when it ends. This link changes the default y-axis timeline. |
| **Logical connections between pre/post process objects set** | | | |
| Logical connection between precondition links. | XOR connection | 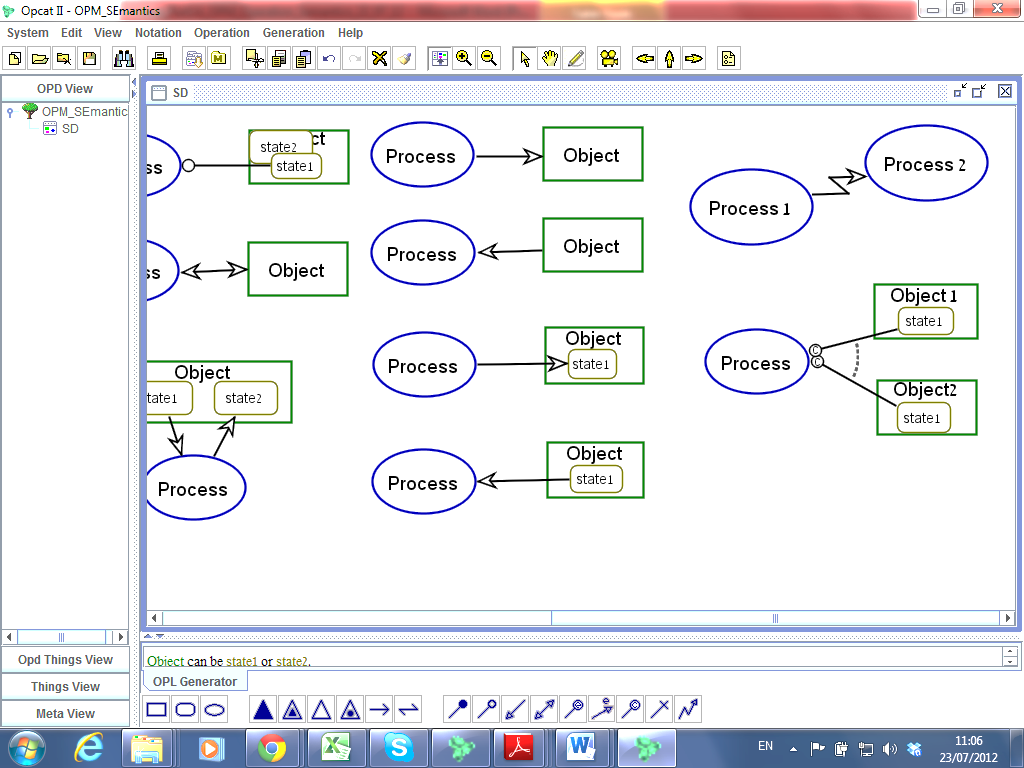 | A connection between process pre/post-conditions object set. Denoting that **any** one of the process pre/post-conditions (in the example, object 1 being at state 1 or object 2 being at state1) is satisfied. |
| Logical connection between precondition links. | OR connection | 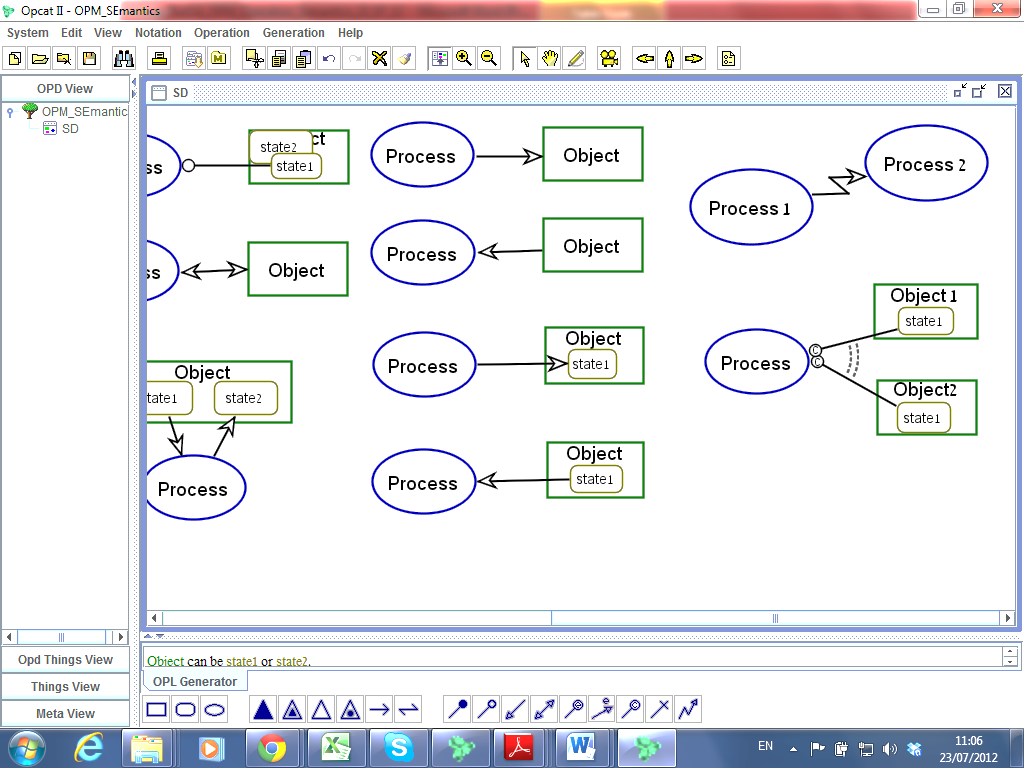 | A connection between process pre/post-conditions object set. Denoting that **exactly** one of the process pre/post-conditions (in the example, object 1 being at state 1 or object 2 being at state1) is satisfied. |
| **Object-Object Structural Links** - Structural hierarchies and characteristic links | | | |
|  | Characterization | 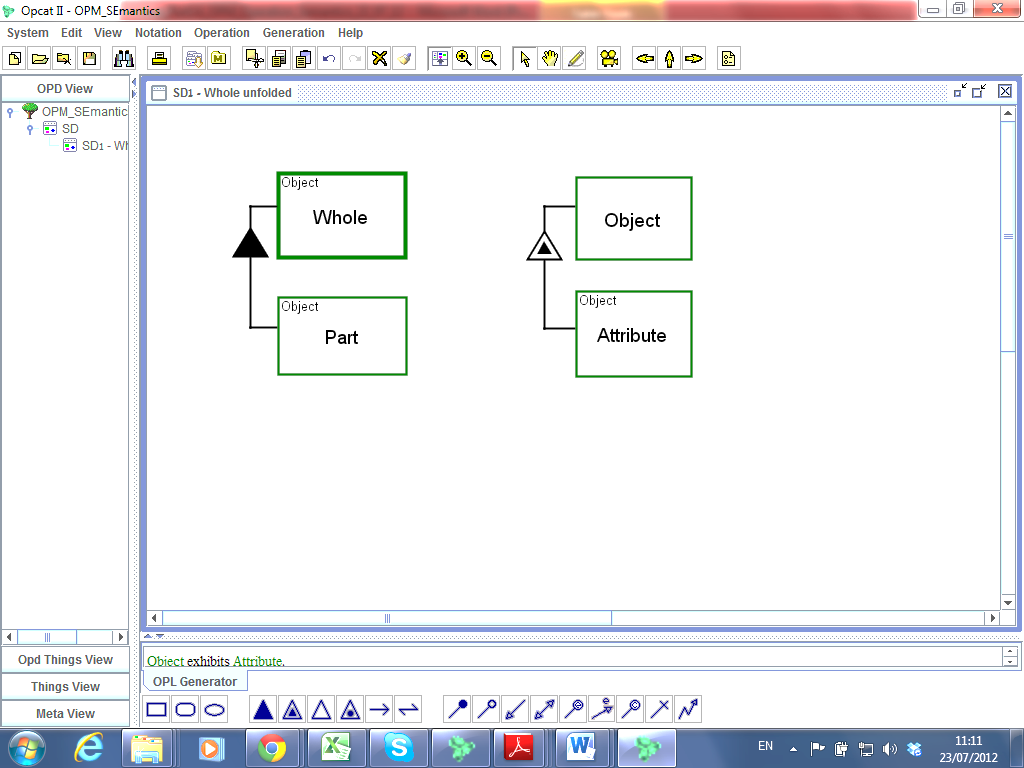 | A fundamental structural relation representing that an element exhibits an attribute object. |
|  | Aggregation | 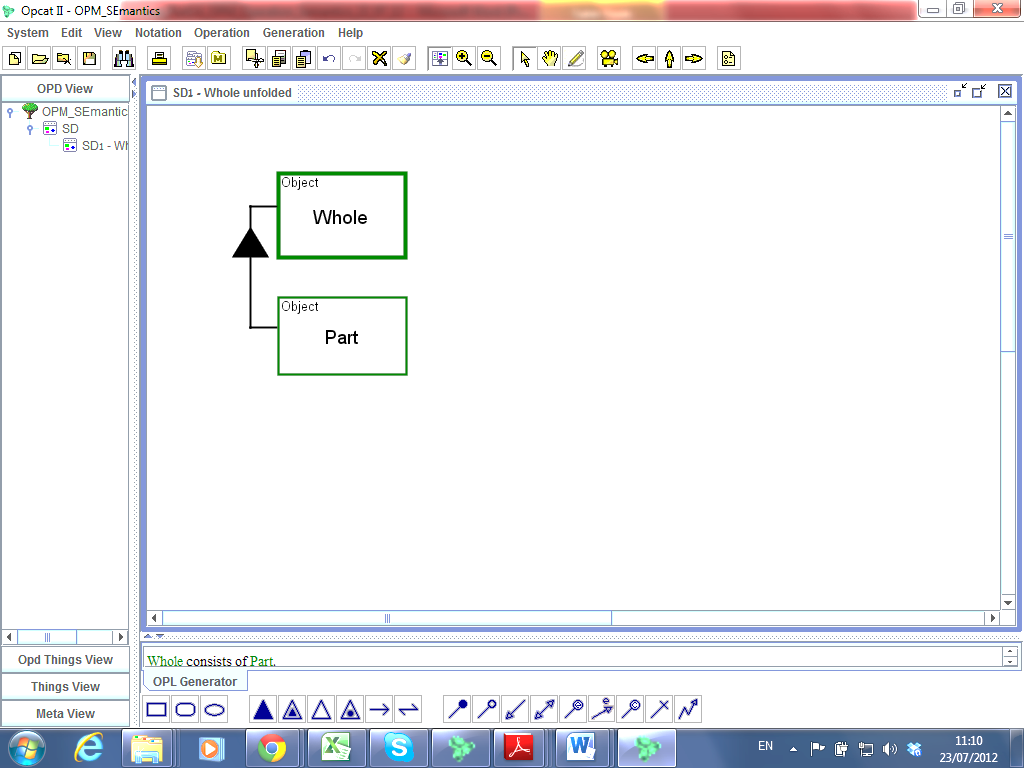 | A fundamental structural relation representing that an object (whole) consists of one or more objects (part(s)). |
|  | General structural link | 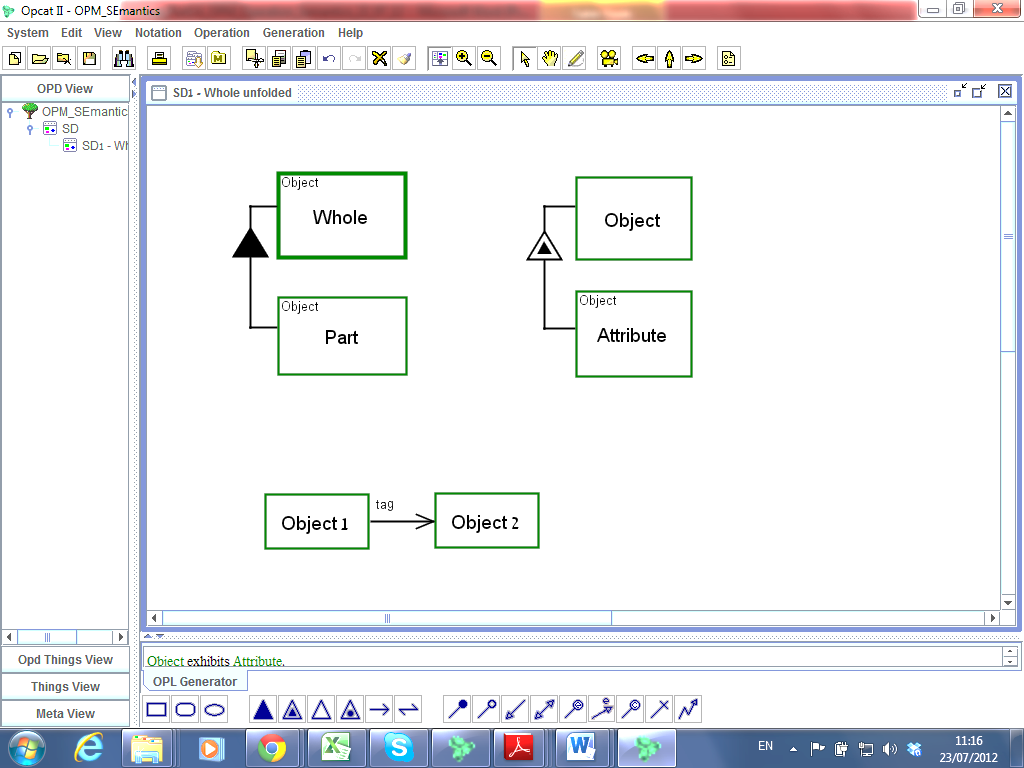 | A unidirectional association between objects that holds for a period of time, possibly with a tag denoting the association semantics |
