# Supplementary material for: Conceptual Model-Based Systems Biology: Mapping Knowledge and Discovering Gaps in the mRNA Transcription Cycle
Source: PLoS One. 2012 Dec 20;7(12):e51430. doi: 10.1371/journal.pone.0051430 (PMC3536069; doi:10.1371/journal.pone.0051430)
Supplement: Table S2 — Transcription model facts and relevant references. (DOCX) [file pone.0051430.s003.docx]

Supplemental Table S2 for

Model-Based Systems Biology:

Mapping Knowledge and Discovering Gaps in the mRNA Transcription Cycle

Judith Somekh^a*^, Mordechai Choder^c^, and Dov Dori^a, b^

^a^Faculty of Industrial Engineering and Management, Technion, Israel Institute of Technology, Haifa 32000, Israel

^b^Engineering Systems Division, Massachusetts Institute of Technology, Cambridge, MA, USA

^c^Faculty of Medicine, Technion, Israel Institute of Technology, Haifa 32000, Israel

Table S2. Transcription facts and relevant references.

| **Ref.** | **Fact/Mechanism** | **Related Molecule** | **No** |
| --- | --- | --- | --- |
| [‎1] | Fcp1 phosphatase activity is stimulated by Rpb4/7 | Fcp1 |  |
| [‎2] | Fcp1 dephosphorylates CTD | Fcp1 |  |
| [‎3] | Rpb4/7 binds Pol II | Rpb4/7, Pol II |  |
| [‎‎4] | TFIIA, TFIID, TFIIH, TFIIE stays on the promoter after transcription initiation | TFIIA, TFIID, TFIIH, TFIIE |  |
| [‎‎4] | TFIIF, TFIIB, Polymerase II disassociated from the promoter after transcription initiation | TFIIF, TFIIB, Polymerase II |  |
| [‎5] | The phosphatase activity of Fcp1 is stimulated by the general transcription factor TFIIF | Fcp1, TFIIF |  |
| [‎‎5] | TFIIB inhibits Fcp1 phosphatase activity | Fcp1, TFIIB |  |
| [‎‎6] | hyperphosphorylated form of the CTD is involved in the interaction with CPSF | CTD, CPSF |  |
| [‎‎7] | TFIIB serine 65 phosphorylation is required after the phosphorylation of serine 5 of RNA pol II C-terminal domain (CTD) has occurred | TFIIB, CTD |  |
| [‎‎7] | TFIIB serine 65 phosphorylation is required before productive transcription initiation begins | TFIIB |  |
| [‎‎8] | Rpb4-Rpb7 complex is not required for stable recruitment of polymerase to active preinitiation complexes | Rpb4/7 |  |
| [‎‎9] | TFIIF and rpb7 has a role in early and late stages of transcription | Rpb7, TFIIF |  |
| [‎3] | RNA polymerase II (Pol II) is composed of ten subunit core and a two subunit dissociable subcomplex, rpb4/rpb7 (comprising the fourth and seventh largest subunits) that form a Heterodimer | Rpb4/7, Pol II |  |
| [‎‎‎3, ‎10, ‎11,  ‎‎12] | Rpb4/7 participate in promoter-directed transcription initiation, elongation and termination | Rpb4/7 |  |
| [‎‎‎13] | FCP1 is a Carboxy-terminal domain (CTD) phosphatase | Fcp1 |  |
| [‎‎‎14] | TFIIF and TFIIB bind the same binding region of fcp1 competitively | Fcp1, TFIIF, TFIIB |  |
| [‎‎‎14] | TFIIF binds Fcp1 at the BRCT domain | Fcp1, TFIIF |  |
| [‎‎‎14] | TFIIB binds Fcp1 at the BRCT domain | Fcp1, TFIIB |  |
| [‎‎‎14] | TFIIF stimulate CTD-phosphatase activity | Fcp1, TFIIB |  |
| [‎‎‎‎2, ‎‎14] | two adjacent regions of yeast Fcp1 containing amino acid residues 457 to 666 and 667 to 732 can each bind yeast RAP74 (subunit of TFIIF) independently, in *S. cerevisiae* | Fcp1, TFIIF |  |
| [‎3] | TFIIF binds Rpb4/7 heterodimer | Rpb4/7, TFIIF |  |
| ‎[‎‎10] | Fcp1/TFIIF/pol II forms a complex | Fcp1/TFIIF/pol II |  |
| [‎‎10, ‎15‎] | Rpb4 recruits the CTD phosphatase Fcp1 | Rpb4, Fcp1 |  |
| [‎‎‎‎16] | amino acid 457 to 666 of Fcp1 binds TFIIB, in *S. cerevisiae* | Fcp1, TFIIB |  |
| [‎‎‎‎16] | amino acid 667 to 732 of Fcp1 binds TFIIB, in *S. cerevisiae* | Fcp1, TFIIB |  |
| [‎‎16] | Fcp1, Phosphatase catalytic domain, named FCPH, is between residues 170 - 363, in *S. cerevisiae* | Fcp1 |  |
| [‎‎‎14] | FCPH catalytic domain of Fcp1 is essential for the function of Fcp1 in vivo | Fcp1 |  |
| [‎‎16] | Fcp1 has a BRCT domain (amino acids 499 – 593 ), in *S. cerevisiae* | Fcp1 |  |
| [‎17, ‎‎18‎] | Serine 2 phosphorylation and dephoshprylation mechanisms:   - Subsequent to ser5 phosphorylation by the action of TFIIH kinase, Ser2 residues are phosphorylated. - phosphorylation of Ser2 by the CDK9 (CDK9 in metazoans/CTDK-I in yeast) subunit of positive-transcription elongation factor b (P-TEFb) activates elongation and RNA processing (splicing and polyadenylation) - Ser2 dephosphorylated after Ser5. - Timing of dephosphorylation is unknown and conjectured to be during termination. | CTD |  |
| [‎‎17, ‎18‎] | Serine 7 phosphorylation and dephoshprylation mechanisms.   - Serine 7 phosphorylation by an unknown kinase occurs during elongation and before splicing. - Timing of dephosphorylation is unknown and conjectured to be during termination. | CTD |  |
| [‎‎17, ‎18‎] | Serine 5 phosphorylation and dephoshprylation mechanisms.   - Ser5 phosphorylated near the 5′ ends of genes depends on the kinase activity of TFIIH. - Phosphorylation on Ser5 by TFIIH helps recruit capping enzymes (capping must be activated after Ser5 phosphorylation). - Ser5 dephosphorylated In yeast and in some mammalian genes toward the 3`end of the transcription unit (after elongation, not clear when exactly). - Ser5 phosphatase activity is implicated in termination. It is not clear when exactly in the transcription cycle the Ser5 phosphatases act. | CTD |  |
| [‎19] | TFIID, TFIIA, stay at the promoter when RNAPII engages in transcript elongation | GTFs |  |

References

1. Kamenski T, Heilmeier S, Meinhart A, Cramer P. Structure and mechanism of RNA polymerase II CTD phosphatases. Mol Cell. 2004;15(3):399-407.
2. Archambault J, Pan G, Dahmus GK, Cartier M, Marshall N, Zhang S, et al. FCP1, the RAP74-interacting subunit of a human protein phosphatase that dephosphorylates the carboxyl-terminal domain of RNA polymerase IIO. J Biol Chem. 1998;273(42).
3. Choder M. Rpb4 and Rpb7: Subunits of RNA polymerase II and beyond. Trends Biochem Sci. 2004;29(12):674-81.
4. Yudkovsky N, Ranish JA, Hahn S. A transcription reinitiation intermediate that is stabilized by activator. Nature. 2000;408(6809):225-9.
5. Friedl EM, Lane WS, Erdjument-Bromage H, Tempst P, Reinberg D. The C-terminal domain phosphatase and transcription elongation activities of FCP1 are regulated by phosphorylation. Proceedings of the National Academy of Sciences. 2003;100(5):2328.
6. Dantonel JC, Murthy KGK, Manley JL, Tora L. Transcription factor TFIID recruits factor CPSF for formation of 3'end of mRNA. Nature. 1997;389(6649):399-402.
7. Wang Y, Fairley JA, Roberts SGE. Phosphorylation of TFIIB links transcription initiation and termination. Current Biology. 2010;20(6):548-53.
8. Orlicky SM, Tran PT, Sayre MH, Edwards AM. Dissociable Rpb4-Rpb7 subassembly of RNA polymerase II binds to single-strand nucleic acid and mediates a post-recruitment step in transcription initiation. J Biol Chem. 2001;276(13):10097-102.
9. Cojocaru M, Jeronimo C, Forget D, Bouchard A, Bergeron D, Cote P, et al. Genomic location of the human RNA polymerase II general machinery: Evidence for a role of TFIIF and Rpb7 at both early and late stages of transcription. Biochem J. 2008;409:139-47.
10. Kimura M, Suzuki H, Ishihama A. Formation of a carboxy-terminal domain phosphatase (Fcp1)/TFIIF/RNA polymerase II (pol II) complex in schizosaccharomyces pombe involves direct interaction between Fcp1 and the Rpb4 subunit of pol II. Mol Cell Biol. 2002;22(5):1577-88.
11. Armache KJ, Mitterweger S, Meinhart A, Cramer P. Structures of complete RNA polymerase II and its subcomplex, Rpb4/7. J Biol Chem. 2005;280(8):7131-4.
12. Sakurai H, Mitsuzawa H, Kimura M, Ishihama A. The Rpb4 subunit of fission YeastSchizosaccharomyces pombe RNA polymerase II is essential for cell viability and similar in structure to the corresponding subunits of higher eukaryotes. Mol Cell Biol. 1999;19(11):7511-8.
13. Ghosh A, Shuman S, Lima CD. The structure of Fcp1, an essential RNA polymerase II CTD phosphatase. Mol Cell. 2008;32(4):478-90.
14. [Kobor MS, Archambault J, Lester W, Holstege FCP, Gileadi O, Jansma DB, et al. An unusual eukaryotic protein phosphatase required for transcription by RNA polymerase II and CTD dephosphorylation in< i> S. cerevisiae</i>. Mol Cell. 1999;4(1):55-62.](http://www.sciencedirect.com/science?_ob=PublicationURL&_tockey=%23TOC%237053%231999%23999959998%23211428%23FLA%23&_cdi=7053&_pubType=J&view=c&_auth=y&_acct=C000228598&_version=1&_urlVersion=0&_userid=10&md5=bdd281fab4ecba4216a629d6c540090f)
15. Kamenski T, Heilmeier S, Meinhart A, Cramer P. Structure and mechanism of RNA polymerase II CTD phosphatases. Mol Cell. 2004;15(3):399-407.
16. Kobor MS, Simon LD, Omichinski J, Zhong G, Archambault J, Greenblatt J. A motif shared by TFIIF and TFIIB mediates their interaction with the RNA polymerase II carboxy-terminal domain phosphatase Fcp1p in saccharomyces cerevisiae. Mol Cell Biol. 2000;20(20):7438-49.
17. Phatnani HP, Greenleaf AL. Phosphorylation and functions of the RNA polymerase II CTD. Genes Dev. 2006;20(21):2922-36.
18. Egloff S, Murphy S. Cracking the RNA polymerase II CTD code. Trends in Genetics. 2008;24(6):280-8.
19. Svejstrup JQ. The RNA polymerase II transcription cycle: Cycling through chromatin. Biochimica et Biophysica Acta (BBA)-Gene Structure and Expression. 2004;1677(1-3):64-73.
